# CD207‐Positive Dendritic Cells Promote Emphysema Through CD8^+^ T Cell Pathway in Chronic Obstructive Pulmonary Disease

**DOI:** 10.1002/advs.202412993

**Published:** 2026-01-07

**Authors:** Shurui Xuan, Yunhui Wu, Feng Liu, Heng Fu, Yaqi Meng, Yingwei Ou, Xijing Yuan, Ian M. Adcock, Man Jia, Xiaoning Zeng, Xin Yao

**Affiliations:** ^1^ Department of Respiratory & Critical Care Medicine the First Affiliated Hospital of Nanjing Medical University Nanjing Jiangsu China; ^2^ Department of Respiratory Medicine Nanjing First Hospital Nanjing Medical University Nanjing Jiangsu China; ^3^ Department of Thoracic Surgery Nanjing Chest Hospital Affiliated Nanjing Brain Hospital Nanjing Medical University Nanjing Jiangsu China; ^4^ Emergency and Critical Care Center Department of Emergency Medicine Zhejiang Provincial People's Hospital (Affiliated People's Hospital Hangzhou Medical College) Hangzhou Zhejiang China; ^5^ Airway Disease Section National Heart and Lung Institute Faculty of Medicine Imperial College London London UK

**Keywords:** birbeck granules, CD207‐positive dendritic cells, CD8^+^ T cells, chronic obstructive pulmonary disease, emphysema, major histocompatibility complex class I molecules

## Abstract

Emphysema remains a major challenge in the management of chronic obstructive pulmonary disease (COPD). This study identifies CD207‐positive dendritic cells (CD207^+^ DCs) as pivotal mediators of emphysema progression. In patients with COPD, the abundance of CD207^+^ DCs in small airways correlates with both emphysema severity and lung function decline (FEV_1_%pred). In a murine emphysema model, adoptive transfer of CD207^+^ DCs reversed the attenuation of emphysema, inflammation and CD8^+^ T‐cell expansion in *CD207*‐knockout mice. Mechanistically, cigarette smoke‐induced epithelial GM‐CSF drives the expansion of CD207^+^ DCs. Upon activation by damage‐associated molecular patterns (DAMPs), these DCs promote CD8^+^ T cell proliferation and activation via Birbeck granule‐mediated MHC‐I antigen cross‐presentation. Collectively, these findings demonstrate that CD207^+^ DCs orchestrate a pathogenic CD8^+^ T‐cell response in emphysema and represent a promising therapeutic target.

## Graphical abstract

CD207^+^ dendritic cells (DCs) drive emphysema by promoting CD8⁺ T cell cytotoxicity via Birbeck granule‐dependent MHC‐I antigen presentation. This DC subset is expanded by cigarette smoke‐induced oxidative stress, which triggers granulocyte‐macrophage colony‐stimulating factor (GM‐CSF) release from airway epithelium. Targeting the CD207⁺ DC‐CD8⁺ T cell axis therefore represents a promising therapeutic strategy for chronic obstructive pulmonary disease (COPD).

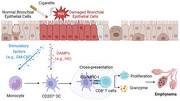

## 1 Introduction

Chronic obstructive pulmonary disease (COPD) is a long‐term lung disorder characterized by irreversible airflow limitation and chronic inflammation of the airways. As a major global health burden, COPD caused approximately 3.5 million deaths in 2021 [1]. Among its key clinical manifestations, emphysema is defined by the permanent enlargement of airspaces distal to the terminal bronchioles, leading to impaired gas exchange [2].

In industrialized countries, cigarette smoke is the leading risk factor for emphysema. Chronic inflammation leads to the loss of alveolar attachments to small airways, which reduces lung elastic recoil [3]. However, the precise pathogenesis remains incompletely defined. Cigarette smoke is known to activate innate immune cells (e.g., macrophages, neutrophils), which in turn promote oxidative stress and protease release [4, 5]. Emerging evidence highlights the critical involvement of adaptive immunity in emphysema progression [6]. CD4^+^ T cells differentiate into various effector subsets, such as Th1, Th2, and Th17, that modulate inflammation and tissue remodeling [7]; B cells facilitate tertiary lymphoid structure formation and humoral responses [8]; and CD8^+^ cytotoxic T lymphocytes (CTLs) induce alveolar apoptosis via the release of granzyme and perforin [9]. Enhanced cytotoxic and pro‐inflammatory activity of CD8^+^ T cells, including increased IFN‐γ production, has been observed in COPD [10], especially in emphysematous tissues [11]. Notably, CD8^+^ T cell‐deficient mice are resistant to cigarette smoke‐induced emphysema, highlighting their pivotal role in pathogenesis [12]. Despite these advances, the precise mechanisms underlying CD8^+^ T cell cytotoxic responses in emphysema remain to be fully elucidated.

Clinically, emphysema should be distinguished from cystic lung diseases, including pulmonary Langerhans cell histiocytosis (PLCH), due to their shared risk factors (e.g., cigarette smoking), overlapping destructive parenchymal changes, similar clinical manifestations such as cough and dyspnea, and impaired lung function [13]. PLCH is characterized by granulomatous lesions with strong histopathological positivity for CD207, a C‐type lectin receptor also known as langerin. CD207 was initially identified in epidermal Langerhans cells [14], then detected in dendritic cells (DCs) across various tissues, including the lung, dermis, and liver [15]. Interestingly, previous studies have reported a selective accumulation of CD207^+^ DCs in COPD patients and current smokers [16]. While DCs are known to play key roles in adaptive immunity in emphysema, the specific contribution of CD207^+^ DCs to disease pathogenesis remains largely unexplored [17]. CD207^+^ DCs have been shown to efficiently mediate antigen presentation and subsequent activation of adaptive immune responses in vivo [18]. Recent evidence indicates that CD207^+^ DCs can promote CD8^+^ T cell activation in settings such as cutaneous vaccine immunogenicity and antiparasitic immunity against *Leishmania* [19, 20]. Based on the parallel features between PLCH and COPD and the existing evidence, we hypothesize that CD207^+^ DCs promote antigen presentation and subsequent activation of CD8^+^ T cell responses to cigarette smoke, thereby contributing to the development of emphysema in COPD.

In this study, we revealed the significant correlations of CD8^+^ T cells and CD207^+^ DCs with emphysema severity and pulmonary function. By combining adoptive transfer with a CD207‐knockout and emphysema mouse model and in vitro co‐culture systems, we investigated the role of CD207^+^ DCs in promoting CD8^+^ T cell expansion and cytotoxicity, and further explored the underlying mechanisms. Additionally, we identified GM‐CSF as a potential regulator of CD207^+^ DC expansion in response to cigarette smoke and oxidative stress exposure. These findings suggest that CD207^+^ DCs are central mediators in the pathogenesis of emphysema in COPD and that they may represent promising targets for immunotherapy.

## 2 Results

### 2.1 CD207^+^ DC Accumulation in Small Airways Correlates with Emphysema Severity and Airflow Limitation in COPD

Current research has identified the increased volume fractions of inflammatory cells in the small airways and lung parenchyma as key contributors to airflow obstruction in patients with COPD [21]. Here, we examined the distribution and abundance of CD8^+^ T cells and CD207^+^ DCs in the small airways and alveolar regions, and further investigated their correlations with the severity of emphysema and pulmonary function in COPD.

**FIGURE 1:**
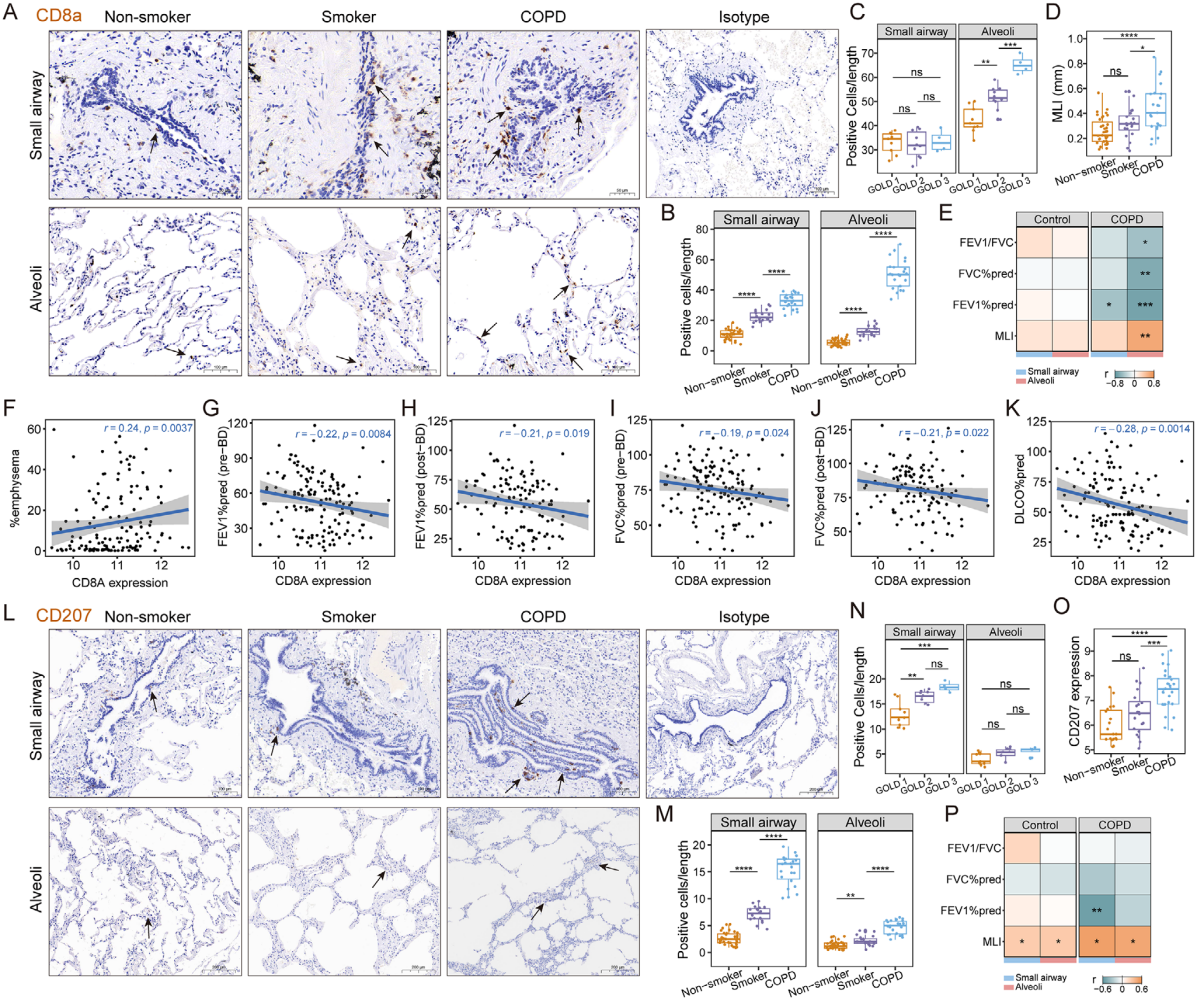
CD8^+^ T cells and CD207^+^ DCs abundance and their associations with emphysema severity and lung function parameters. A) Representative immunohistochemistry (IHC) images for CD8^+^ T cells in small airways and alveolar parenchyma from non‐smokers (*n* = 35), smokers (*n* = 20) and COPD (*n* = 23) in our cohort. Scale bars are shown in each panel. Original magnification, 300× for small airway regions; 200× for alveolar regions and isotype control. B) Quantification of CD8^+^ T cell density (cells/mm) in small airways (normalized to basement membrane length) and alveolar regions (normalized to alveolar septum length), across non‐smokers, smokers and COPD samples. C) CD8^+^ T cell density in the small airways and alveolar regions of COPD patients, stratified by GOLD stage 1, 2, and 3. D) Quantification of alveolar mean linear intercept (MLI) in non‐smoker, smoker, and COPD groups. E) Correlation heatmap of CD8^+^ T cell density in small airways and alveolar regions with alveolar MLI and pulmonary function parameters in our cohort. (F–K) Analysis of a public bulk lung tissue gene expression dataset (GSE47460, *n* = 145). F) Association of *CD8A* gene expression with per cent emphysema (%emphysema, defined as the percentage of lung pixels less than −950 Hounsfield units). G‐K) Correlation analysis between *CD8A* gene expression and lung function indicators: G) pre‐bronchodilator (BD) FEV1%pred; H) post‐BD FEV1%pred; (I) pre‐BD FVC%pred; (J) post‐BD FVC%pred; (K) DLCO%pred. L) Representative IHC images of CD207^+^ DC in small airways and alveolar parenchyma from non‐smoker, smoker, and COPD samples in our cohort. Scale bars are indicated within each panel. Original magnification, 300× for small airway regions; 200× for alveolar regions and isotype control. M) Quantification of CD207^+^ DC density, standardized to basal membrane length or septum length (cells/mm) in small airways and alveolar regions from non‐smoker, smoker, and COPD samples. N) CD207^+^ DC density in small airways and alveolar regions among GOLD stage 1, 2, and 3 COPD patients. O) *CD207* gene expression from pooled public datasets of small airways (GSE5058 and GSE8545) from non‐smokers (*n* = 19), smokers (*n* = 21), and COPD patients (*n* = 21). P) Correlation analysis between CD207^+^ DC density in small airways and alveolar regions with alveolar MLI and pulmonary function parameters in our cohort. Data are presented as box‐and‐whisker plots showing all points. ^*^ *p* < 0.05, ^**^ *p* < 0.01, ^***^ *p* < 0.001, ^****^ *p* < 0.0001. Statistical significance was determined by one‐way ANOVA with Tukey's post hoc test (B‐D, M‐O) or Spearman correlation analysis (E‐K, P).

We conducted a retrospective analysis of lung tissues from 35 non‐smoker controls (never‐smokers without airflow limitation, 20 smoker controls (current‐smokers without airflow limitation, and 23 COPD patients (classified as GOLD stage 1 [*n* = 9], stage 2 [*n* = 10], and stage 3 [*n* = 4], Table 1 and Table 2)). Immunohistochemistry (IHC) showed increased CD8^+^ cell density in both small airways and alveolar regions in COPD patients compared to the non‐smoker and smoker controls (Figure 1A,B). COPD patients with GOLD stage 2 and 3 had significantly higher alveolar CD8^+^ T cell levels than those with GOLD stage 1, while no significant differences were observed in the small airways (Figure 1C). We assessed the severity of emphysema by measuring the alveolar mean linear intercept (MLI). As expected, COPD patients exhibited significantly larger alveolar MLI than non‐smoker and smoker controls (Figure 1D). Correlations of CD8^+^ T cell density in small airways and lung parenchyma with disease severity (including alveolar MLI and pulmonary function parameters) were evaluated across COPD and control subgroups (Figure 1E). This analysis revealed a strong positive correlation between alveolar CD8^+^ T cell levels and MLI, and negative correlations with percentage of predicted forced expiratory volume in 1 s (FEV1%pred), percentage of predicted forced vital capacity (FVC%pred) and FEV1/FVC in COPD, while these correlations were not statistically significant in the control group.

**TABLE 1:** Clinical characteristics of non‐smoker, smoker, and COPD individuals in our cohort.

	Non‐smoker	Smoker	COPD	p
Number (n)	35	20	23	
Age (year)	61.54 ± 9.91	58.00 ± 9.56	60.22 ± 8.70	0.415
Sex (M/F)	21/14	18/2	20/3	0.0192
BMI (kg/m^2^)	23.85 ± 3.03	23.66 ± 2.59	22.56 ± 2.05	0.184
Smoking index (pack‐years)	0	32.42 ± 15.03	31.09 ± 17.58	<0.001
FEV1/FVC	85.00 ± 5.23	82.03 ± 6.05	61.77 ± 10.05	<0.001
FEV1%pred	99.84 ± 8.17	93.16 ± 11.47	74.82 ± 20.66	<0.001
FVC%pred	101.53 ± 10.92	105.08 ± 15.66	85.87 ± 17.54	<0.001

**TABLE 2:** Clinical characteristics of COPD Subgroups.

	GOLD stage 1	GOLD stage 2	GOLD stage 3	p
Number (n)	9	10	4	
Age (year)	56.56 ± 5.94	60.20 ± 9.21	68.50 ± 8.66	0.067
Sex (M/F)	7/2	8/2	4/0	1
BMI (kg/m^2^)	22.47 ± 2.02	22.87 ± 2.12	21.99 ± 2.38	0.775
Smoking index (pack‐years)	30.00 ± 17.32	31.00 ± 20.79	33.75 ± 12.50	0.944
FEV1/FVC	68.01 ± 5.87	58.85 ± 10.04	55.02 ± 11.83	0.038
FEV1%pred	95.79 ± 10.68	67.89 ± 6.24	44.95 ± 6.40	<0.001
FVC%pred	92.54 ± 14.44	85.83 ± 20.08	70.92 ± 7.61	0.12

We analyzed *CD8A* (encoding the CD8α chain of CD8^+^ T cells) gene expression using the transcriptomic dataset GSE47460 from 145 COPD patients (details in Table S1). *CD8A* expression was significantly positively correlated with percent emphysema (%emphysema, percentage of lung pixels less than −950 Hounsfield units, Figure 1F), and negatively correlated with key lung function parameters, including pre‐bronchodilator (BD) FEV1%pred (Figure 1G), post‐BD FEV1%pred (Figure 1H), pre‐BD FVC%pred (Figure 1I), post‐BD FVC%pred (Figure 1J), and the diffusing capacity of the lungs for carbon monoxide percentage predicted (DLCO% pred, Figure 1K).

Our findings validated that CD8^+^ T cell infiltration is significantly associated with the severity of airflow limitation and emphysematous changes in COPD, indicating that CD8^+^ T cells may serve as valuable biomarkers and potential intervention targets. However, the precise mechanisms of CD8^+^ T cell cytotoxic responses and the interplay between CD8^+^ T cells and other immune cell populations in the progression of emphysema remain to be elucidated. To further investigate our hypothesis, the expression and localization of CD207^+^ DCs were examined in our cohort. Quantitative analysis for IHC demonstrated a significantly higher abundance of CD207^+^ DCs in COPD patients compared to non‐smoker and smoker controls, especially in the small airways (Figure 1L,M). Stratified analysis showed that CD207^+^ DCs in the small airways were significantly increased in patients with more severe disease (GOLD stages 2 and 3) compared to those in the GOLD stage 1 group (Figure 1N).

The transcriptomic levels of CD207 were further analyzed in small airway epithelium samples from publicly available datasets (GSE5058 and GSE8545), including normal non‐smokers (*n* = 19), normal smokers (*n* = 21), and COPD patients (*n* = 21). Consistent with our protein‐level findings, CD207 gene expression was significantly elevated in COPD patients compared to non‐smoker and smoker controls (Figure 1O). Correlation analysis showed that CD207^+^ DC levels were positively associated with alveolar MLI in both COPD and control groups in our cohort (Figure 1P). Notably, the density of CD207^+^ DCs in the small airways exhibited a significant negative correlation with FEV1%pred in COPD patients.

Our results demonstrated a marked accumulation of CD207^+^ DCs within the small airways of COPD patients, which was closely associated with both emphysema severity and reduced pulmonary function. These findings highlight a potential role of CD207^+^ DCs in the progression of emphysema and the development of airflow limitation in COPD.

### 2.2 CD207^+^ DCs Drive Experimental Emphysema and Associated Pulmonary Inflammation

**FIGURE 2:**
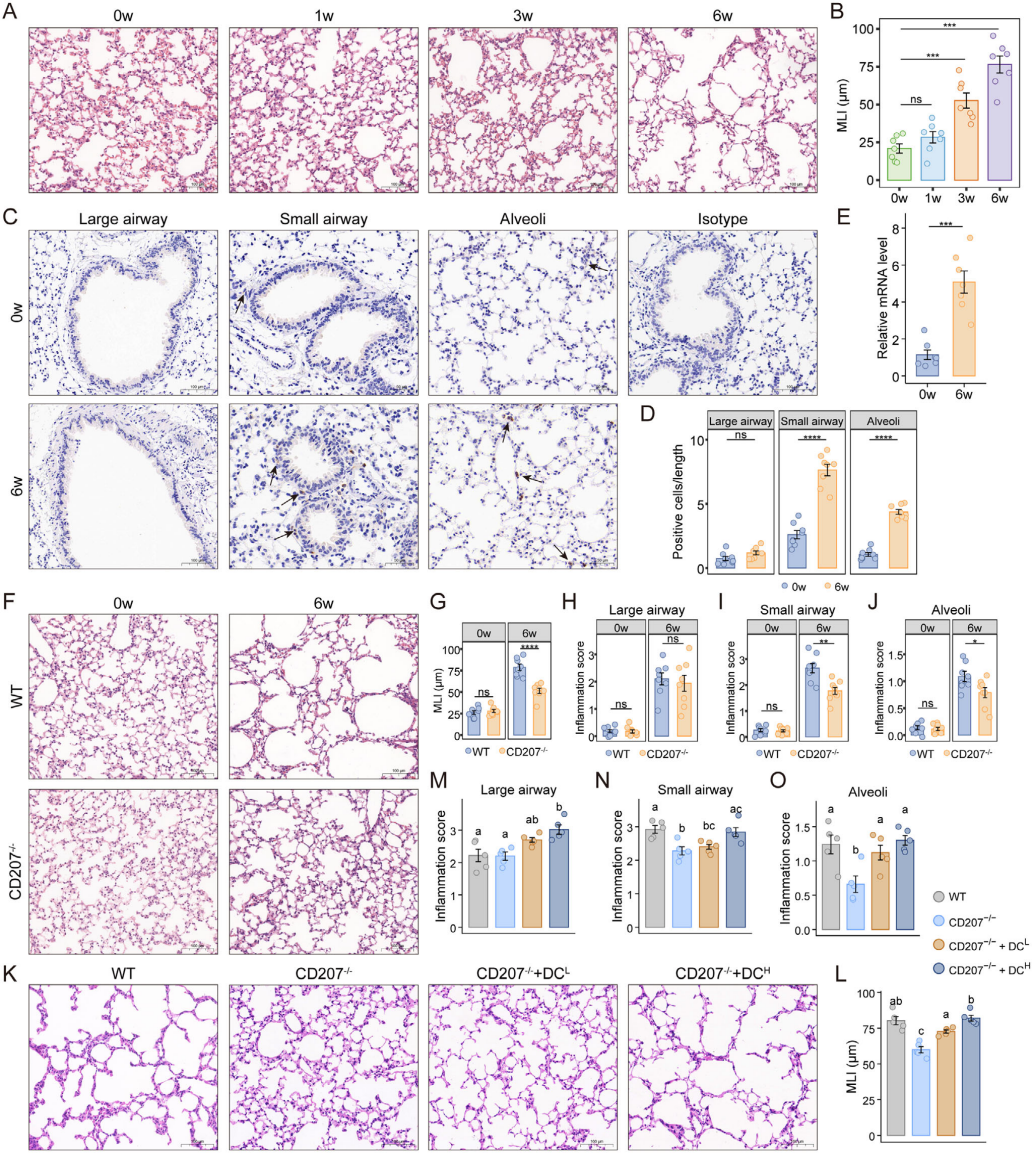
Ablation of CD207^+^ DCs alleviates experimental emphysema and local inflammation, while their adoptive transfer restores pathology. A) Representative hematoxylin and eosin (H&E) staining of lung parenchyma from mice exposed to O_3_ for 0, 1, 3, and 6 weeks (*n* = 7 for each group) in the emphysema models. Scale bar, 100 µm. Original magnification, 200×. B) Comparison of alveolar MLI in the four groups. C) Representative immunohistochemistry for CD207 in large airways, small airways, and alveolar parenchyma of control (0w) and emphysema (6w) mice (*n* = 8 per group). Scale bars are indicated within each panel. Original magnification: 200× (large airways), 400× (small airways), 300× (alveolar regions), 250× (isotype control). D) Quantification of CD207^+^ cells in the indicated lung regions of control and emphysema groups. E) Relative *CD207* mRNA expression in whole lung homogenates from control and emphysematous mice. F) Representative H&E staining of wild‐type (WT) and CD207 knockout (*CD207^−/−^*) mice following 0 and 6 weeks of O_3_ exposure. Scale bar, 100 µm. Original magnification, 200×. G) Comparison of alveolar MLI between WT and *CD207^−/−^* mice after 0 and 6 weeks O_3_ exposure. H–J) Quantification of inflammation scores in the large airways (H), small airways (I), and alveolar parenchyma (J) for WT and *CD207^−/−^* mice after 0 and 6 weeks O_3_ exposure. K) Representative images of H&E staining lung sections from WT, *CD207^−/−^*, and *CD207^−/−^* mice that received an adoptive transfer of low‐dose (*CD207^−/−^* + DC^L^) or high‐dose (*CD207^−/−^* + DC^H^) CD207^+^ DCs after 6 weeks O_3_ exposure. Scale bar, 100 µm. Original magnification, 200×. L) Quantification of alveolar MLI across the four experimental groups (*n* = 5 per group). M‐O) Quantification of inflammation scores in large airways (M), small airways (N), and alveolar parenchyma (O) for the four groups. Data are shown as mean ± SEM. ^*^ *p* < 0.05, ^**^ *p* < 0.01, ^***^ *p* < 0.001, ^****^ *p* < 0.0001. Different letters (a, b, c) indicate statistically significant differences between groups (p < 0.05). Statistical significance was determined by one‐way ANOVA with Dunnett's post hoc test (B), two‐tailed Student's *t*‐test (D, E, G‐J), or one‐way ANOVA with Tukey's post hoc test for multiple comparisons (L‐O).

Tobacco smoke induces localized pulmonary inflammation and oxidative stress, disrupting the oxidant‐antioxidant balance and serving as a major driver of COPD development. Notably, emerging evidence indicates that emphysema progression can continue even after smoking cessation [22], suggesting that persistent oxidative stress may contribute to ongoing lung injury in emphysema [23]. Ozone (O_3_) exposure has previously been reported to be an effective inducer of emphysema [24], thus we employed this model to investigate the role of CD207^+^ DCs in emphysema pathogenesis in this study. Eight‐week‐old wild‐type (WT) mice were exposed to O_3_ (3 ppm, 3 h per day) for 6 weeks to induce emphysema, which was assessed by measuring alveolar MLI (Figure 2A,B). Compared to controls (0‐week O_3_ exposure), emphysema mice (6‐week O_3_ exposure) showed a significant increase in CD207^+^ DC density in both small airways and alveolar regions, with the most prominent infiltration in the small airways (Figure 2C,D). Moreover, CD207 mRNA expression in lung tissue was significantly upregulated in the emphysema models (Figure 2E).

To further investigate the role of CD207^+^ DCs, we established an emphysema model in CD207 knockout (*CD207^−/−^*) mice. After 6‐week O_3_ exposure, *CD207^−/−^* mice exhibited significantly attenuated alveolar enlargement (Figure 2F,G) and lower inflammation scores in the small airways (Figure 2I) and alveolar regions (Figure 2J) compared to WT controls, while no significant difference was observed in the large airways (Figure 2H). Immunofluorescence analysis of MUC5AC (a marker of airway mucus secretion) and Occludin (a key tight junction protein) expression in airways of *CD207^−/−^* and WT mice revealed that *CD207* deficiency had no significant effect on the expression of either marker compared to WT mice (Figure S1). This suggests that the protective effect of *CD207* deficiency against emphysema is not attributable to alterations in airway mucus production or epithelial barrier integrity, but is more likely associated with the modulation of immune cell activity and inflammatory responses.

We performed adoptive transfer of purified CD207^+^ DCs isolated from bone marrow‐derived dendritic cells (BMDCs) into *CD207^−/−^* mice at low (5 × 10^4^ cells) and high (1 × 10^5^ cells) doses. Transfer efficiency was confirmed by flow cytometric analysis of pulmonary CD207^+^ DC frequencies (Figure S2). Following the adoptive transfer of CD207^+^ DCs, *CD207^−/−^* mice exhibited significantly exacerbated emphysema (Figure 2K,L). Notably, the high‐dose transfer resulted in more severe alveolar destruction compared to the low‐dose group. Furthermore, compared to the *CD207^−/−^* group, these high‐dose recipients also displayed more pronounced immune infiltration, with significantly elevated inflammation scores across the large airways, small airways, and alveolar regions (Figure 2M–O).

These results demonstrate that CD207^+^ DCs accumulate in the small airways and alveolar regions of emphysematous lungs. While genetic deficiency of CD207 alleviates both emphysema severity and local inflammation, the adoptive transfer of these cells restores pathology. Collectively, these findings establish that CD207^+^ DCs contribute to emphysema development and emphasize the need for further investigation into their pathogenic mechanisms.

### 2.3 CD207^+^ DCs Facilitate CD8^+^ T Cell Expansion and Granzyme Expression in Emphysema

**FIGURE 3:**
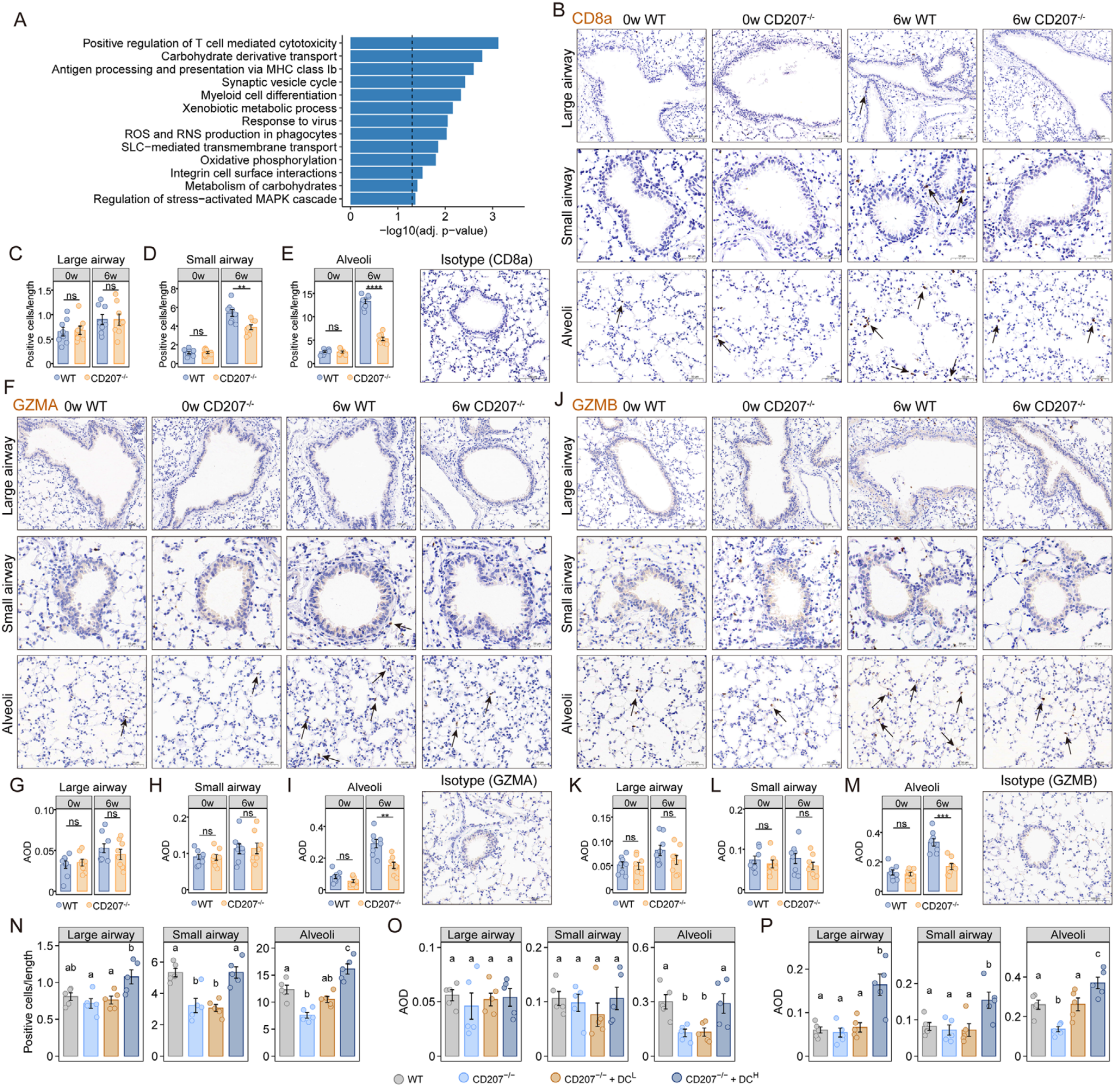
Association of CD207^+^ DCs with CD8^+^ T cell abundance and granzyme expression in O_3_‐induced emphysema model. A) Pathway enrichment analysis of genes significantly correlated with *CD207* expression (*p* < 0.05) in lung tissues from COPD patients in the GSE47460 dataset. *P*‐values were adjusted using FDR correction. B) Representative immunohistochemical staining for CD8α in large airways, small airways and alveolar regions of WT and *CD207^−/−^* mice after 0 and 6 weeks of O_3_ exposure. Scale bars are shown in each panel. Original magnification: 200× (large airways), 400× (small airways), 300× (alveolar regions), 250× (isotype control). C–E) Quantification of CD8α‐positive cells normalized to basement membrane or alveolar septa length (cells/mm) in each region. F) Representative immunohistochemical staining for GZMA in large airways, small airways and alveolar regions of WT and *CD207^−/−^* emphysema mouse model. Scale bars are indicated in each panel. Original magnification: 200× (large airways), 400× (small airways), 300× (alveolar regions), 250× (isotype control). G‐I) Quantification of GZMA expression in each region using average optical density (AOD). J) Representative immunohistochemistry for GZMB in large airways, small airways and alveolar regions of WT and *CD207^−/−^* mice. Scale bars are indicated in each panel. Original magnification: 200× (large airways), 400× (small airways), 300× (alveolar regions), 250× (isotype control). K–M) Quantification of GZMB expression by AOD in each region. Data are presented as mean ± SEM. ^**^ *p* < 0.01, ^***^ *p* < 0.001, ^****^ *p* < 0.0001. N–P) The four experimental groups: WT, *CD207^−/−^*, and *CD207^−/−^* mice that received an adoptive transfer of low‐dose (*CD207^−/−^* + DC^L^) or high‐dose (*CD207^−/−^* + DC^H^) CD207^+^ DCs were subjected to 6 weeks of O_3_ exposure. N) Quantification of CD8α‐positive cell density (cells/mm). Cell counts were normalized to the length of the basement membrane (for airways) or alveolar septa (for parenchyma). O,P) Quantification of GZMA (O) and GZMB (P) expression measured by AOD. Data are shown as mean ± SEM. ^**^ *p* < 0.01, ^***^ *p* < 0.001, ^****^ *p* < 0.0001. Different letters (a, b, c) indicate statistically significant differences between groups (*p* < 0.05). Statistical significance was determined by two‐tailed Student's t‐test (C‐E, G‐I, K‐M), or one‐way ANOVA with Tukey's post hoc test for multiple comparisons (N‐P).

CD207‐associated differentially expressed genes identified in lung tissues from COPD patients in the GSE47460 dataset were significantly enriched in pathways regulating T cell cytotoxicity, and antigen processing and presentation via MHC class Ib molecules (Figure 3A). These results suggest the potential involvement of CD207 in CD8^+^ T cell‐mediated immunity in COPD.

In addition to CD8^+^ T cells, the CD207^+^ DC subset may also influence adaptive immunity involving CD4^+^ T cells and B cells. Therefore, we quantified CD4^+^ T cell (using CD4 as a marker) and B cell (CD19 as a marker) densities in large airways, small airways, and alveolar regions of the *CD207^−/−^* emphysema model. No significant differences in CD4^+^ (Figure S3A–D) or CD19^+^ cell (Figure S3E–H) densities were observed between *CD207^−/−^* and WT mice in any lung region.

Given these pathway associations, we next assessed CD8^+^ T cell (CD8α as a marker) abundance in different lung compartments of our mouse model (Figure 3B). Emphysematous WT mice exhibited a marked expansion of CD8^+^ T cells in both the small airways and alveolar parenchyma, whereas this increase was significantly attenuated in *CD207^−/−^* mice (Figure 3C–E). Furthermore, we examined the expression of cytotoxic effectors granzyme A (GZMA, Figure 3F) and granzyme B (GZMB, Figure 3J). Both effectors were predominantly upregulated in the alveolar regions, rather than in the large and small airways. Consistently, *CD207^−/−^* mice showed significantly reduced GZMA (Figure 3G–I) and GZMB (Figure 3K–M) expression in the alveolar regions, indicating impaired cytotoxic responses.

In the adoptive transfer experiments, the transfer of CD207^+^ DCs reversed the reduction in CD8^+^ T‐cell expansion (Figure S4A; Figure 3N) and the expression of the granzymes GZMA (Figure S4B; Figure 3O) and GZMB (Figure S4C; Figure 3P) observed in *CD207^−/−^* mice. Specifically, the high‐dose transfer led to a significant increase in both CD8^+^ T cells and GZMB expression in all three compartments, coupled with a marked increase in GZMA expression within the alveolar tissue.

In summary, our findings demonstrate that *CD207* deficiency selectively reduced CD8^+^ T cell accumulation and the expression of cytotoxic effector molecules in the alveolar regions, with minimal effects on CD4^+^ T cell or B cell infiltration. Adoptive‐transfer rescue experiments provide direct evidence for the indispensable role of CD207^+^ DCs in driving CD8^+^ T‐cell activation and granzyme expression in vivo during emphysema pathogenesis.

### 2.4 Heparan Sulfate‐Activated CD207^+^ DCs Promote CD8^+^ T Cell Proliferation and Cytotoxic Responses

In the pathogenesis of emphysema, ongoing oxidative stress induced by cigarette smoking leads to structural cell damage and the release of damage‐associated molecular patterns (DAMPs) [25], such as high mobility group box 1 (HMGB1), which can serve as antigens for DC activation [26]. Recent studies have shown that heparan sulfate (HS) has a high affinity for CD207 [27], suggesting that both HMGB1 and HS may act as potential endogenous ligands through which CD207^+^ DCs modulate CD8^+^ T cell responses.

**FIGURE 4:**
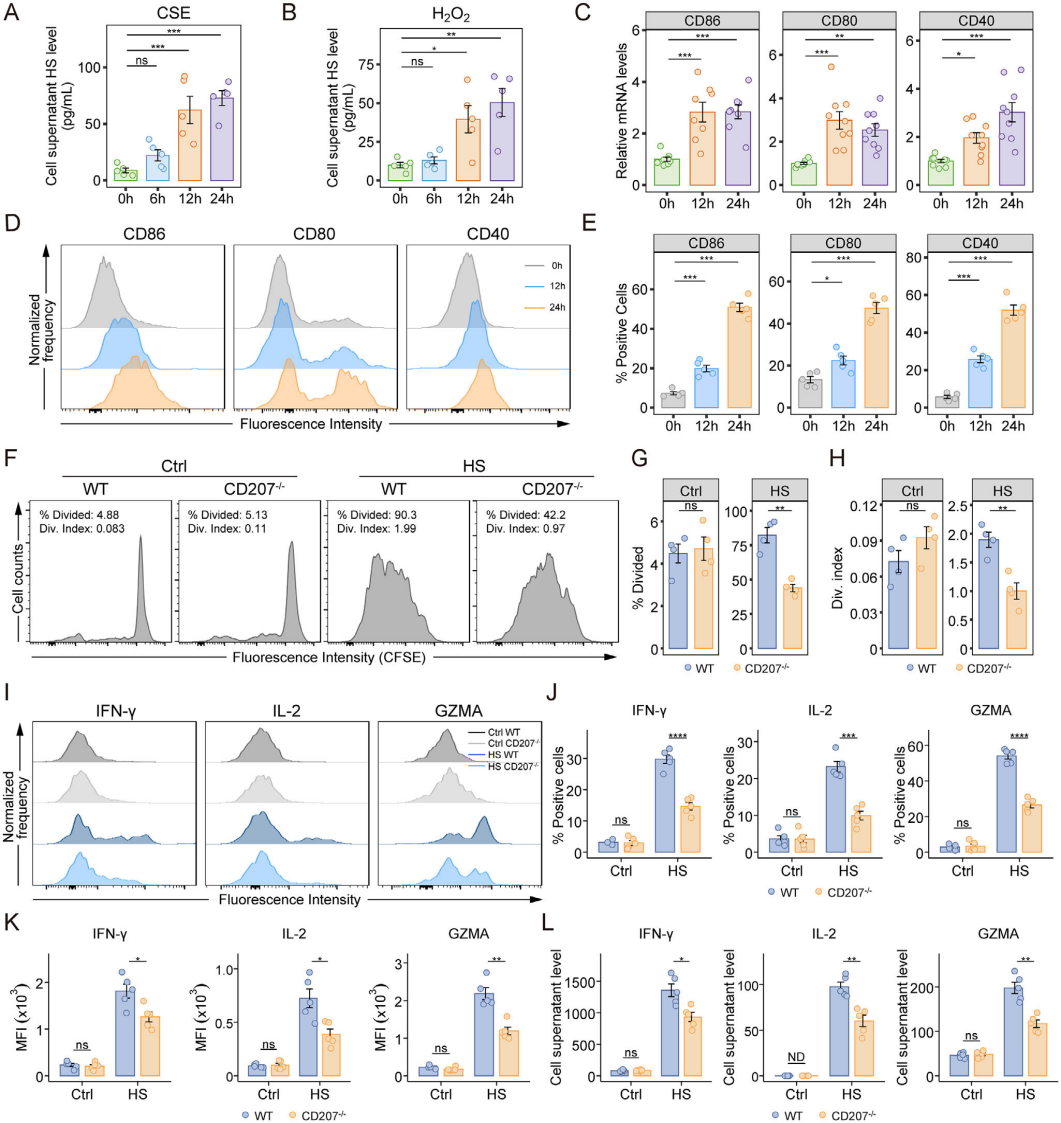
*CD207* deficiency in DCs impairs CD8^+^ T Cell proliferation and cytotoxicity in vitro. A) Heparan sulfate (HS) levels measured by ELISA in the supernatant of 16HBE cells treated with 5% CSE for 0, 6, 12, and 24 h (*n* = 5 per group). B) HS level in supernatants of 16HBE cells treated with 100 µM H_2_O_2_ at the indicated time points (*n* = 5 per group). C) Relative mRNA expression of *CD86*, *CD80*, and *CD40* in CD207^+^ DCs treated with HS for 0, 12, and 24 h (*n* = 8–9 per group). D) Representative flow cytometry histograms showing fluorescence intensity of CD86, CD80, and CD40 in CD207^+^ DCs at the indicated time points (*n* = 5 per group). E) Frequency of CD86^+^, CD80^+^, and CD40^+^ cells among CD207^+^ DCs (thresholds defined by Fluorescence Minus One [FMO] controls). F–H) Naïve CD8^+^ T cells were labeled with carboxyfluorescein diacetate succinimidyl ester (CFSE) and co‐cultured with BMDCs from WT and *CD207^−/−^* mice with or without HS stimulation for 48 h (*n* = 4 per group). F) Representative histograms showing CFSE dilution in CD8^+^ T cells. G, H) Percentage of divided CFSE‐labeled cells (% Divided) and average number of divisions per cell among all cells in the culture (division index, Div. index), as quantified by FlowJo software. I–L) CD8^+^ T cells were co‐cultured with HS‐stimulated WT and *CD207^−/−^* BMDCs for 48 h (*n* = 5 per group). I) Representative flow cytometry histograms of IFN‐γ, IL‐2 and GZMA fluorescence intensity in CD8^+^ T cells. J) Percentage of IFN‐γ‐, IL‐2‐ and GZMA‐positive cells in CD8^+^ T cells (thresholds defined by FMO controls). K) Mean fluorescence intensity (MFI) of IFN‐γ, IL‐2 and GZMA in CD8^+^ T cells. L) Concentrations of IFN‐γ, IL‐2 and GZMA in culture supernatants. Data are presented as mean ± SEM. ^*^ *p* < 0.05, ^**^ *p* < 0.01, ^***^ *p* < 0.001, ^****^ *p* < 0.0001. Statistical significance was determined using a one‐way ANOVA with Dunnett's post‐hoc test (A‐C, E) or a two‐tailed Student's t‐test (G, H, J‐L).

Human bronchial epithelial cells (16HBE) were exposed to 5% cigarette smoke extract (CSE) and 100 µM hydrogen peroxide (H_2_O_2_) to simulate cigarette smoke and oxidative stress in vitro. These treatments significantly increased the HS (Figure 4A,B) and HMGB1 (Figure S5A,B) secretion in a time‐dependent manner. CD207^+^ DCs were isolated from bone marrow‐derived dendritic cells (BMDCs) using magnetic beads and stimulated with exogenous heparan sulfate (HS) or HMGB1. HS treatment significantly upregulated the co‐stimulatory molecules CD86, CD80, and CD40 at both the mRNA (RT‐qPCR, Figure 4C) and protein (flow cytometry, Figure 4D,E) levels in CD207^+^ DCs. In contrast, HMGB1 selectively increased CD80 and CD40 protein expression (Figure S5D,E), without affecting CD86 protein levels, and did not significantly alter the mRNA expression of any of the three molecules (Figure S5C). As CD207^+^ DCs are classified as a type 1 conventional DC (cDC1) subset, we further examined the activation of cDC1 and cDC2 subsets in murine BMDCs to assess the specificity of HS stimulation (Figure S5F). HS increased co‐stimulatory molecule expression in the broader cDC1 population, though this effect was less pronounced than in purified CD207^+^ DCs (Figure S5G). In cDC2s, HS stimulation resulted in a significant increase in CD80 expression, while CD86 and CD40 levels remained unchanged (Figure S5H).

To further elucidate the role of CD207^+^ DCs in modulating CD8^+^ T cell responses, we established a mixed lymphocyte reaction (MLR) system by co‐culturing carboxyfluorescein diacetate succinimidyl ester (CFSE)‐labeled naïve CD8^+^ T cells with BMDCs from WT and *CD207^−/−^* mice, followed by HS stimulation. CD8^+^ T cells exhibited markedly reduced proliferation when co‐cultured with *CD207^−/−^* BMDCs (Figure 4F), as indicated by both a lower percentage of divided cells (%Divided, Figure 4G) and a decreased division index (Figure 4H). Moreover, CD8^+^ T cells co‐cultured with *CD207^−/−^* BMDCs showed significantly reduced expression of effector cytokines (IFN‐γ and IL‐2) and GZMA after HS stimulation, as evidenced by lower frequencies of positive cells (Figure 4I,J), reduced mean fluorescence intensity (MFI, Figure 4K), and consistently diminished secretion in culture supernatants (Figure 4L). Together, these findings highlight that CD207 is indispensable for the ability of DCs to drive the effective expansion and cytotoxic response of CD8^+^ T cells.

To determine whether *CD207* deletion affects the composition of DC subsets, we analyzed the frequencies and absolute numbers of cDC1 and cDC2 populations in both lung tissue and BMDCs (detailed gating strategy shown in Figure S5I). No significant differences in the frequencies or absolute counts of cDC1 and cDC2 subsets were observed between WT and *CD207^−/^* ^−^ mice in either lung tissue (Figure S5J,K) or BMDCs (Figure S5L,M), indicating that *CD207* deficiency does not disrupt DC subset distribution. These results suggest that CD207^+^ DCs promote CD8^+^ T cell cytotoxic responses through a functional rather than a quantitative mechanism.

### 2.5 Disruption of Birbeck Granules in CD207^+^ DCs Compromises Major Histocompatibility Complex Class I Trafficking and CD8^+^ T Cell Priming

We next examined whether the activation status of WT and *CD207^−/−^* BMDCs in the co‐culture system would influence CD8^+^ T cell proliferation and cytotoxicity. No significant differences were observed in the expression of co‐stimulatory molecules CD86, CD80, and CD40 at either the mRNA (Figure S6A–C) or protein levels (Figure S6D,E) between WT and *CD207^−/−^* BMDCs. To further determine whether *CD207* deficiency selectively impairs the maturation of DC subsets and subsequent T cell activation, we compared the expression of these molecules on cDC1 and cDC2 subsets. The results showed that *CD207* knockout did not alter the activation status of either subset (Figure S6D,F,G). These findings suggest that CD207^+^ DC‐mediated CD8^+^ T cell activation occurs independently of co‐stimulatory molecule expression and may rely on alternative mechanisms of T cell activation.

**FIGURE 5:**
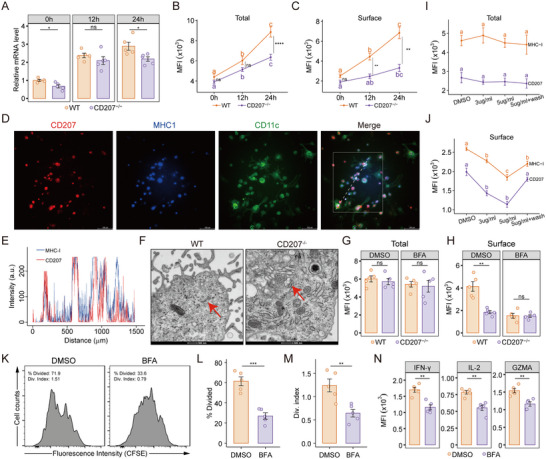
Disruption of Birbeck granules (BGs) in CD207^+^ DCs impaired MHC‐I trafficking to the cell surface and suppressed CD8^+^ T cell responses. A‐C) WT and CD207^−/−^ BMDCs were treated with HS (10 µg/ml) for 0, 12, and 24 h. A) Relative mRNA expression levels of MHC‐I were quantified via RT‐qPCR and normalized to GAPDH expression. Total (B) and surface (C) MFI of MHC‐I expression in BMDCs were measured by flow cytometry. D) Representative immunofluorescence images of CD207 (red), MHC‐I (blue) and CD11c (green) in WT BMDCs. Scale bar, 100 µm. E) Fluorescence pixel intensity plot of MHC‐I and CD207 for the dashed line across WT BMDCs. F) Representative TEM images of WT and *CD207^−/−^* BMDCs. Red arrows point to BGs. Scale bar: 500 nm. G,H) WT and *CD207^−/−^* BMDCs were stimulated with HS for 12 h, followed by treatment with BFA (3 µg/mL) or DMSO vehicle control for 3 h. Total (G) and surface (H) MFI of MHC‐I expression in BMDCs were subsequently analyzed. I,J) CD207^+^ DCs were isolated from BMDCs using magnetic bead sorting and treated with BFA at 3 µg/mL or 5 µg/mL for 3 h, 5 µg/mL BFA followed by a 3‐h washout, or DMSO vehicle control. Total (I) and surface (J) MFI of CD207 and MHC‐I expression in CD207^+^ DCs. K‐N) CD207^+^ DCs were stimulated with HS for 24 h, treated with BFA (5 µg/ml) or DMSO as a control for 3 h, and then co‐cultured with CFSE‐labeled naïve CD8^+^ T cells for 48 h. K) Representative flow cytometry histograms showing CFSE fluorescence decay in CD8^+^ T cells. L,M) Quantification of % Divided (L) and Div. index (M) were calculated from CFSE dilution using FlowJo software. N) MFI of IFN‐γ, IL‐2 and GZMA in CD8^+^ T cells in the co‐culture. Data are presented as mean ± SEM. ^*^ *p* < 0.05, ^**^ *p* < 0.01, ^***^ *p* < 0.001. Different letters (a, b, c) indicate statistically significant differences between groups (*p* < 0.05). Statistical significance was determined using a one‐way ANOVA with Tukey's post‐hoc test (B, C, I, J), or a two‐tailed Student's *t*‐test (A‐C, G, H, L‐N).

Therefore, we assessed the expression of major histocompatibility complex class I (MHC‐I), a critical molecule for antigen presentation to CD8^+^ T cells. The murine MHC‐I protein (H‐2K) and its corresponding gene (*H2‐K1*) expression in co‐cultures were analyzed by flow cytometry and RT‐qPCR, respectively. At both baseline and after 24 h of HS treatment, *CD207^−/−^* BMDCs exhibited significantly lower MHC‐I mRNA expression compared to WT controls (Figure 5A). While HS stimulation induced upregulation of both total and surface MHC‐I protein (measured by MFI) in WT and *CD207^−/−^* BMDCs, this increase was markedly attenuated in *CD207^−/−^* cells. Notably, the reduction in surface MHC‐I expression on *CD207^−/−^* BMDCs was more pronounced and occurred earlier than the decrease observed in total MHC‐I (Figure 5B,C). This indicates that CD207^+^ DC subset is essential for optimal MHC‐I upregulation and surface presentation in response to HS stimulation, potentially explaining the impaired CD8^+^ T cell activation observed in the *CD207^−/−^* models.

Immunofluorescence analysis showed overlapping fluorescence patterns of MHC‐I and CD207 in cellular regions of interest (Figure 5D,E), indicating potential co‐localization or functional interplay. Birbeck granules (BGs) are key organelles involved in intracellular trafficking and antigen degradation following CD207‐dependent uptake and internalization of HIV‐1 antigens, and their formation is closely linked to CD207 [28]. As described by Ray et al., BGs participate in CD207 intracellular recycling: surface CD207 is internalized into early endosomes, dynamically localized to BGs, and subsequently recycled back to the plasma membrane. BGs strongly colocalized and connected with the endosomal recycling compartment (ERC), indicating they are a specialized subdomain of the ERC [29]. Interestingly, the intracellular pathway of MHC‐I during cross‐presentation shares a similar recycling trajectory. MHC‐I is internalized into early endosomes, accumulates in ERC, and then presents peptide antigens on the cell surface [30]. This parallel led us to hypothesize that BGs may orchestrate MHC‐I trafficking and cross‐presentation in CD207^+^ DCs, though the underlying molecular mechanisms remain poorly understood.

Transmission electron microscopy (TEM) revealed morphological abnormalities in BGs from *CD207^−/−^* BMDCs, including shortening, deformation, and curvature, along with unevenly thickened bilayers and a loss of internal electron‐dense structures (Figure 5F). Previous studies have shown that Brefeldin A (BFA) induces the fusion of BGs with other components of the endocytic pathway, thereby inhibiting membrane trafficking [29]. Compared to the vehicle control, BFA treatment eliminated the difference in surface MHC‐I expression between WT and *CD207−/‐* BMDCs after 12 h of HS treatment (Figure 5H), with no significant changes observed in total cellular MHC‐I levels (Figure 5G). To investigate the intracellular effects of BG inhibition, CD207^+^ DCs were sorted from BMDCs and treated with BFA at 5 µg/mL, an intermediate concentration of 3 µg/mL, or vehicle control. In parallel, a washout group was established following 5 µg/mL BFA exposure. Surface and total cellular expression levels of CD207 and MHC‐I were subsequently quantified. BFA treatment led to comparable reductions in surface CD207 and MHC‐I expression, both of which were restored after washout (Figure 5J), while total cellular protein levels remained unchanged (Figure 5I). These results establish BGs as crucial transporters of CD207 and implicate their role in MHC‐I intracellular trafficking and surface localization. Functionally, BG disruption in CD207^+^ DCs significantly impaired their ability to drive CD8^+^ T cell proliferation (Figure 5K–M) and led to decreased intracellular levels of IFN‐γ, IL‐2, and GZMA in co‐culture assays (Figure 5N).

Collectively, these findings demonstrate that CD207^+^ DCs facilitate MHC‐I transport via BGs, thereby promoting efficient antigen cross‐presentation and robust CD8^+^ T cell responses.

### 2.6 GM‐CSF Promotes CD207^+^ DC Expansion via Differentiation, Proliferation, and Early Survival

Previous studies have reported the potential role of GM‐CSF [31], IL‐4 [32], IL‐34 [33], and TGF‐β [34] in CD207^+^ DCs and Langerhans cell development. We therefore assessed their capacity to induce CD207^+^ DCs from bone marrow‐derived monocytes in vitro.

**FIGURE 6:**
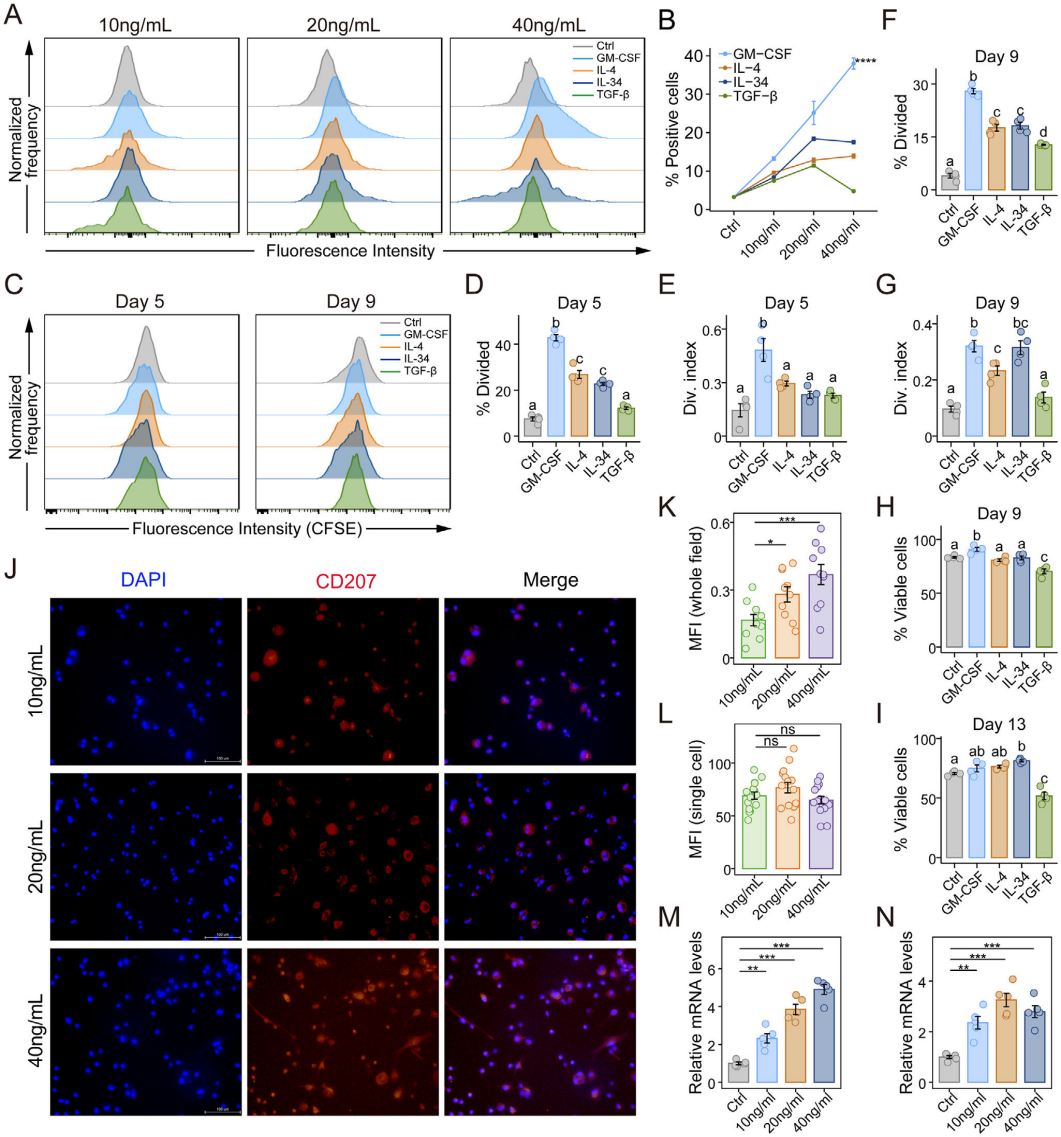
GM‐CSF promotes CD207^+^ DC differentiation, proliferation, and early survival. A, B) Bone marrow‐derived monocytes were induced as baseline control, and were stimulated with GM‐CSF, IL‐4, IL‐34, or TGF‐β at concentrations of 10, 20, and 40 ng/mL, respectively. A) Representative histograms of CD207 fluorescence intensity in BMDCs detected by flow cytometry. B) Quantification of the proportion of CD207^+^ DCs across different concentrations of GM‐CSF, IL‐4, IL‐34 and TGF‐β. C‐G) CFSE‐labeled CD207^+^ DC proliferation was tracked at days 3–5 and 7–9 of BMDC differentiation with GM‐CSF, IL‐4, IL‐34, or TGF‐β stimulation (20 ng/mL each). C) Representative histograms showing CFSE fluorescence decay in CD207^+^ DCs at day 5 and day 9. D–G) Quantification of % Divided and Div. index at day 5 (D, E) and day 9 (F, G) were calculated from CFSE dilution using FlowJo software. H,I) Proportion of CD207^+^ DC survival was assessed at day 9 (H) and day 13 (I) of BMDC differentiation culture. (J) Representative immunofluorescence images of CD207^+^ DCs stimulated with GM‐CSF at concentrations of 10, 20, and 40 ng/mL: CD207 in red, DAPI in blue. K) CD207 MFI measured per whole field under different GM‐CSF concentrations. L) CD207 MFI quantified per single cell under different GM‐CSF concentrations. M,N) Relative gene expression levels of *Spi1* (encoding PU.1; M) and *Runx3* (N) in monocytes were measured by RT‐qPCR. Data are presented as mean ± SEM. ^*^ *p* < 0.05, ^**^ *p* < 0.01, ^***^ *p* < 0.001, ^****^ *p* < 0.0001. Different letters (a, b, c) indicate statistically significant differences between groups (*p* < 0.05). Statistical significance was determined by two‐way ANOVA with Tukey's post‐hoc test (B), one‐way ANOVA with Tukey's post‐hoc test (D‐I), or one‐way ANOVA with Dunnett's post‐hoc test (K‐N).

Flow cytometry analysis revealed that GM‐CSF significantly increased the frequency of CD207^+^ DCs within BMDCs in a concentration‐dependent manner, compared to IL‐4 and IL‐34. While low concentrations of TGF‐β increased the proportion of CD207^+^ DCs and higher concentration led to a reduction (Figure 6A,B, detailed gating strategy and fluorescence minus one [FMO] controls shown in Figure S7A,B). To determine whether the increased proportion was attributable to proliferative or survival advantages, we further assessed the proliferation and survival of CD207^+^ DCs. CFSE dilution assays were performed during mid‐phase (days 3–5, Figure 6D,E) and late‐phase (days 7–9, Figure 6F,G) of BMDC differentiation, with analysis conducted on days 5 and 9, respectively (Figure 6C). The results indicated that GM‐CSF, IL‐34, and IL‐4 significantly promoted CD207^+^ DC proliferation across both timepoints. Survival assessment showed that GM‐CSF initially enhanced viability on day 9 (Figure 6H), but this effect diminished by day 13 (Figure 6I), suggesting a short‐term effect in promoting CD207^+^ DC survival, with limited long‐term efficacy. IL‐34 exhibited a delayed pro‐survival effect with a significant increase in viability observed at later time points, while TGF‐β treatment impaired CD207^+^ DC viability at both examined time points.

Immunofluorescence analysis validated that GM‐CSF increased CD207^+^ DC abundance (Figure 6J,K) but did not alter CD207 surface expression levels on individual cells (Figure 6L), suggesting its primary role in driving cellular expansion rather than modulating CD207 expression at the single‐cell level. The generation of CD207^+^ DCs is promoted by transcription factors, such as PU.1 and Runt‐related transcription factor 3 (Runx3) [35]. GM‐CSF stimulation significantly upregulates PU.1 (*Spi1*, Figure 6M) and Runx3 (*Runx3*, Figure 6N) mRNA expression in monocytes, indicating a role in promoting CD207^+^ DC differentiation.

Overall, our findings demonstrate that GM‐CSF significantly increases the abundance of CD207^+^ DCs by promoting differentiation, along with enhancing proliferation and early survival. This coordinated regulation underscores the critical role of GM‐CSF in the development and homeostasis of CD207^+^ DCs.

### 2.7 Smoking and Oxidative Stress Increase GM‐CSF Production in Airway Epithelial Cells

**FIGURE 7:**
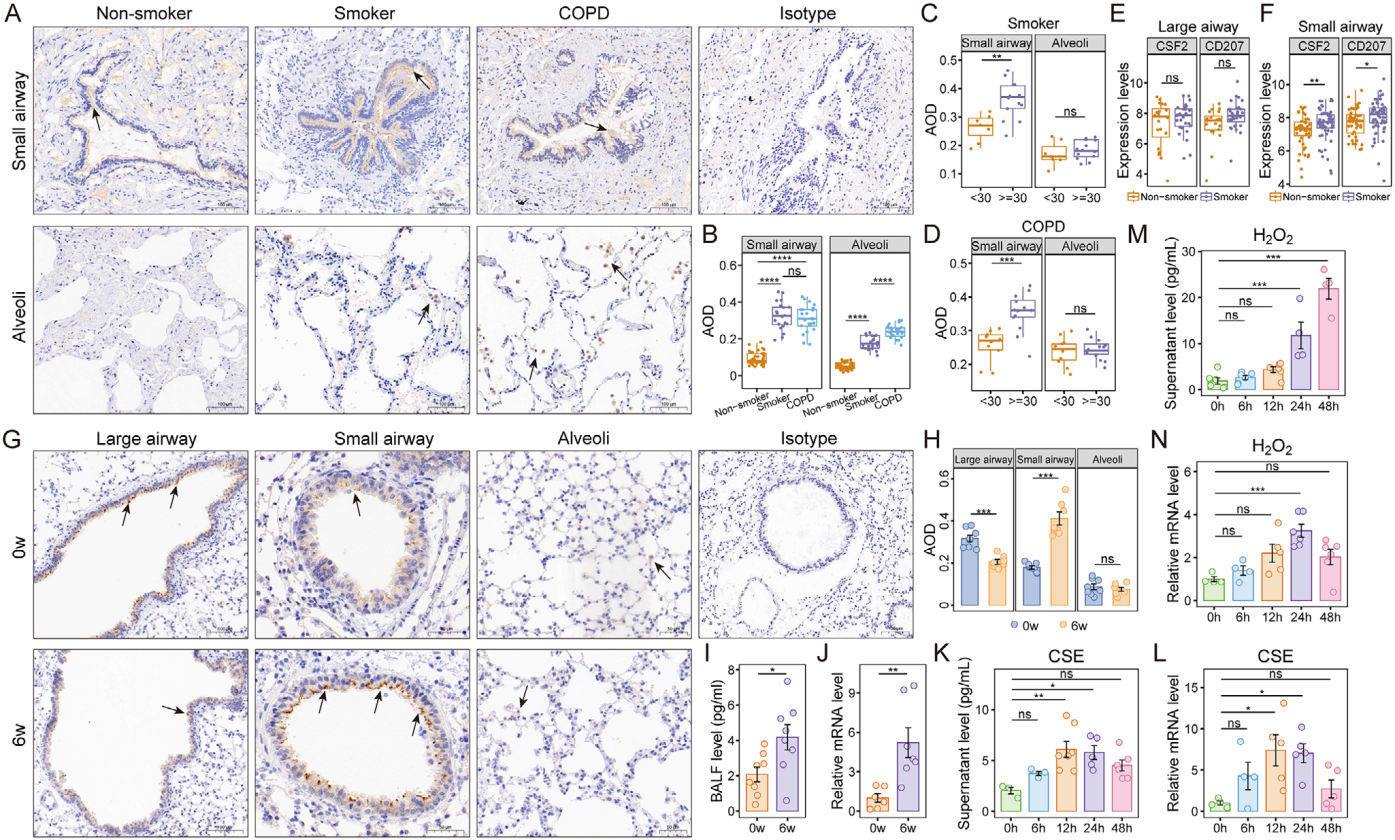
Elevated GM‐CSF is induced by smoking‐related stressors. A) Representative IHC staining for GM‐CSF in the small airways and alveolar parenchyma of our cohort. Scale bars are indicated within each panel. Original magnification, 200× for small airway regions; 200× for alveolar regions and isotype control. B) Quantification of GM‐CSF staining in small airways and alveolar parenchyma by AOD values. C, D) GM‐CSF expression in smoking controls (C) and COPD patients (D) stratified by smoking index (< 30 vs. ≥ 30 pack‐years). E,F) Gene expression levels of GM‐CSF (*CSF2*) and *CD207* from large airway (E) and small airway (F) epithelium in non‐smokers and smokers in the GSE18385 dataset. G) Representative IHC staining for GM‐CSF in lung tissue of control and emphysema mice exposed to O_3_ for 0 and 6 weeks, respectively. Scale bars are indicated within each panel. Original magnification: 200× (large airways), 400× (small airways), 300× (alveolar regions), 150× (isotype control). H) Quantification of GM‐CSF staining in each region of each group by AOD values. I) GM‐CSF protein levels in BALF as measured by ELISA. J) GM‐CSF mRNA expression in lung tissue of model mice was measured by RT‐qPCR. K, L) 16HBE cells were treated with 5% CSE for 0, 6, 12, 24, and 48 h, respectively: (K) GM‐CSF level in cell supernatant; (L) GM‐CSF mRNA expression relative to GAPDH in 16HBE cells. M,N) 16HBE cells were treated with 100 µm H_2_O_2_ for 0, 6, 12, 24, and 48 h, respectively: (M) GM‐CSF level in cell supernatant; (N) GM‐CSF mRNA expression relative to GAPDH in 16HBE cells. Data are presented as mean ± SEM. ^*^ *p* < 0.05, ^**^ *p* < 0.01, ^***^ *p* < 0.001, ^****^ *p* < 0.0001. Statistical significance was determined by one‐way ANOVA with Tukey's post hoc test for multiple comparisons (B), a two‐tailed Student's *t*‐test (C, D, H‐J), Wilcoxon rank sum test (E‐F), or one‐way ANOVA with Dunnett's post hoc test (K‐N).

Given the observed increase of CD207^+^ DCs in COPD, and the critical role of GM‐CSF in promoting CD207^+^ DC expansion, we assessed whether GM‐CSF expression is elevated in patients with COPD and whether its levels are associated with established disease risk factors. IHC demonstrated that GM‐CSF staining was significantly increased in the small airways and alveolar regions of both smoker controls and COPD patients compared to non‐smokers (Figure 7A,B). When stratified by smoking history, GM‐CSF expression was significantly higher in the small airways of smoker controls with a ≥30 pack‐year history compared to those with a <30 pack‐year history. No significant difference was observed in the lung parenchyma between the two groups (Figure 7C). A similar trend was observed in COPD patients (Figure 7D). Analysis of the GSE18385 dataset, comprising human large and small airway samples, further showed significantly elevated mRNA levels of GM‐CSF and *CD207* in the small airways of smokers compared to non‐smokers (Figure 7F), whereas no significant difference was observed in the large airways between these groups (Figure 7E).

In the murine emphysema model, we observed a marked upregulation of GM‐CSF expression in the small airways of O_3_‐exposed mice compared with controls, whereas no significant change was detected in the alveolar regions. Interestingly, GM‐CSF expression was decreased in the large airways following O_3_ exposure (Figure 7G,H). In addition, GM‐CSF protein levels were significantly elevated in bronchoalveolar lavage fluid (BALF) of O_3_‐exposed mice (Figure 7I), and GM‐CSF mRNA expression in whole lung tissue was also markedly increased (Figure 7J).

We exposed 16HBE14o‐ human bronchial epithelial cells (16HBE) to 5% cigarette smoke extract (CSE) and 100 µM hydrogen peroxide (H_2_O_2_) in vitro, thereby modeling key environmental risk factors for COPD to further elucidate the mechanisms underlying GM‐CSF upregulation in response to smoking‐related and oxidative stress stimuli. Both treatments led to a pronounced increase in GM‐CSF mRNA expression (Figure 7L,N), as measured by RT‐qPCR, and significantly elevated GM‐CSF release into the culture supernatant (Figure 7K,M), as determined by ELISA. These results demonstrate that GM‐CSF production by airway epithelial cells is robustly induced by smoking‐associated and oxidative stressors, implicating the bronchial epithelium as a key source of GM‐CSF in the pathogenic response to COPD risk factors.

## 3 Discussion

In this study, we demonstrated elevated CD207^+^ DCs and CD8^+^ T cells in the small airways and alveolar parenchyma of COPD patients compared to both non‐smoker and smoker controls. CD207^+^ DC density exhibited a positive correlation with alveolar MLI and a negative correlation with FEV1%pred. These findings were further supported in an O_3_‐induced emphysema mouse model, in which *CD207* deletion attenuated emphysema and led to reduced CD8^+^ T cell infiltration and lower expression of GZMA and GZMB. Mixed lymphocyte reaction assays confirmed that the absence of CD207^+^ DCs impaired the proliferation and activation of CD8^+^ T cells. Mechanistically, functional disruption of BGs suggested that CD207^+^ DCs facilitate MHC‐I trafficking through BGs to enhance antigen cross‐presentation to CD8^+^ T cells. In addition, we identified GM‐CSF as a key regulator coordinating the differentiation, proliferation, and early survival of CD207^+^ DCs. This pathway was further activated by cigarette smoke and oxidative stress in human bronchial epithelial cells, indicating an immunoregulatory mechanism that promotes CD207^+^ DC accumulation and function in emphysema progression.

We focused on the CD207^+^ DC subset in this study based on both clinical and immunological considerations. Strong CD207 expression in PLCH‐associated cystic lesions, together with the overlap in risk factors, clinical features, and emphysematous changes between PLCH and COPD, prompted us to explore the role of CD207^+^ DCs in emphysema pathogenesis. While our study emphasizes this specific subset, we do not exclude the potential complementary roles of other DC populations or innate immune cells, such as macrophages and neutrophils, in tissue destruction and immune dysregulation in emphysema [36]. Importantly, our findings highlight a distinct cellular axis that warrants further mechanistic investigation. Future studies are needed to clarify the interactions among these immune cell populations within the lung microenvironment and to define their respective contributions to disease progression.

In our study, TEM analysis showed that BGs in *CD207^−/−^* BMDCs displayed structural abnormalities, consistent with previous reports highlighting the essential role of CD207 in BG formation in epidermal Langerhans cells [29]. Disruption of BGs with Brefeldin A eliminated differences in surface MHC‐I expression between WT and *CD207^−/−^* BMDCs, without affecting total protein levels. In sorted CD207^+^ DCs, BFA treatment reduced surface CD207 and MHC‐I expression in a reversible manner, with no change in total protein. These results indicate that BGs are important transporters for CD207 and are involved in MHC‐I surface presentation. Functionally, BG disruption impaired the ability of CD207^+^ DCs to promote CD8^+^ T cell proliferation and cytotoxicity. However, previous work suggested that *CD207* deficiency disrupts BGs without substantially affecting antigen presentation via class I or class II pathways [37]. This discrepancy may be due to several factors. First, while the previous study focused on epidermal Langerhans cells, our research investigated CD207^+^ DCs from lung tissue and BMDCs. Recent evidence indicates that LCs are a distinct type of mononuclear phagocyte (MNP) derived from the yolk sac, which has a lineage distinct from that of DCs [38]. Second, the studies utilized different antigens and experimental conditions: the previous work employed protein antigens (such as OVA) and microbial components, whereas we used the glycosaminoglycan heparan sulfate, which engages different endocytic receptors and intracellular trafficking pathways [39, 40]. Thus, variations in cell type, antigen properties, and experimental context may account for the observed differences.

Long‐term exposure to irritant gases or particulate matter, especially from cigarette smoke, is a major cause of alveolar damage [41]. Excessive oxidative stress from these exposures leads to cellular injury and death [42], which can persist even after smoking cessation [43]. Oxidative stress induces the fragmentation of lung elastin, loss and breakdown of the extracellular matrix (ECM) and the release of DAMPs, such as HS and HMGB1, from damaged cells [44, 45]. These DAMPs can activate the innate immune cells (e.g., macrophages, DCs, eosinophils, and natural killer cells) through binding to pattern recognition receptors (PRRs) [46]. The C‐type lectin receptor CD207 binds mannose and related carbohydrates via its carbohydrate recognition domain in a calcium‐dependent manner [47], and has a high affinity for HS [48]. Our experiments demonstrate that CSE and oxidative stressors (H_2_O_2_) induce the secretion of HS and HMGB1 by bronchial epithelial cells. Furthermore, exogenous HS activated CD207^+^ DCs and promoted the proliferation and activation of naïve CD8^+^ T cells in a mixed lymphocyte reaction co‐culture system. These findings suggest that the persistent oxidative stress and unresolved inflammation may amplify pathological cascades in vivo by sustaining DAMP release and immune activation. Nevertheless, cigarette‐associated antigens also play a significant role in activating adaptive immunity within the inflammatory environment of emphysema [49]. A systematic comparison of the effects of CSE and oxidative stress on antigen production and the downstream immune activation in our model will be important for elucidating the mechanisms underlying emphysema progression.

Cigarette smoking and oxidative stress induce local inflammatory responses in the bronchial epithelium, leading to the release of various cytokines that drive the differentiation of bone marrow precursor cells into DCs and promote their accumulation in lung tissue [50]. In addition, these factors may also alter the balance of DC subsets, favoring the expansion of pro‐inflammatory populations such as myeloid DCs (mDCs) while reducing regulatory DCs [51]. Our study demonstrates that GM‐CSF plays a pivotal role in mediating these effects. We found that exposure to cigarette smoke and oxidative stress elevate GM‐CSF levels, which in turn upregulate key transcription factors involved in CD207^+^ DC differentiation, promote cellular proliferation, and support cell survival. Notably, the pro‐survival effect of GM‐CSF is most pronounced in the early stage and appears to decrease over time, indicating that its primary role is to support the initial expansion of CD207^+^ DCs rather than their long‐term maintenance. This transient effect suggests that GM‐CSF helps maintain an immune balance by supporting an early response to injury while limiting excessive DC accumulation, which may be disrupted by chronic cigarette smoking and oxidative stress. Overall, our findings highlight the central role of GM‐CSF in coordinating the differentiation, proliferation, and early survival of CD207^+^ DCs. These insights offer potential avenues to target dysregulated DC responses in chronic airway inflammation. Moreover, our data reveal that IL‐34 enhances the frequency of the CD207^+^ DC subset in BMDCs in a concentration‐dependent manner. IL‐34 promoted the proliferation of CD207^+^ DCs and enhanced their survival at later time points, although its overall effect was less pronounced than that of GM‐CSF. As IL‐34 is a cytokine that signals through the CSF‐1R receptor [52], these results highlight its previously unrecognized role in supporting the expansion and maintenance of CD207^+^ DCs. Given that immune responses associated with risk factors such as smoking and oxidative stress are influenced by multiple factors, IL‐34 may represent a novel and promising regulator worthy of further investigation. In contrast to the sustained expansion driven by GM‐CSF, IL‐4 and IL‐34, TGF‐β initially increased the proportion of CD207^+^ DCs, followed by a subsequent decline at a later time point. TGF‐β exposure led to a decrease in viable CD207^+^ DCs, suggesting elevated apoptosis, consistent with previous reports [53]. In addition, previous studies indicate that TGF‐β promotes the differentiation of tolerogenic DCs, leading to a shift in DC subset balance [54]. Both increased cell death and changes in subset composition may therefore contribute to the decline in CD207^+^ DCs observed with TGF‐β treatment. Elucidating the precise mechanisms underlying the effect of TGF‐β will be important for understanding how the lung immune environment is regulated under different cytokine signals.

In our animal model, we observed a significant increase in CD207^+^ DCs and CD8^+^ T cells in both the small airways and alveolar regions, rather than in the large airways. Notably, CD207^+^ DCs were prominently concentrated in the small airways, along with elevated GM‐CSF expression, whereas CD8^+^ T cells were highly enriched in the alveolar regions. These spatial distribution patterns were consistent with those seen in human lung samples. In addition, our murine model showed increased markers of CD4^+^ T cells and B cells in the alveolar regions. However, B cell density in the large and small airways remained unchanged, while CD4^+^ T cells exhibited a trend of an increase in both airway compartments. The selective accumulation of GM‐CSF and CD207^+^ DCs in the small airways, together with the overlapping spatial localization of CD207^+^ DCs and CD8^+^ T cells in the small airways and alveolar regions, suggests that smoke exposure amplifies local GM‐CSF/CD207 signaling cascades and further supports the hypothesis of a potential functional interaction between CD207^+^ DCs and CD8^+^ T cells in driving emphysema pathogenesis. Overall, these findings suggest that the small airways may act as a primary site of inflammation and mediator production in emphysema, whereas the alveolar parenchyma serves as the main site of effector cell accumulation. The large airways may be more closely associated with CD4^+^ T cell‐mediated immune regulation. The distinct localization of these adaptive immune cells highlights their specific roles in disease pathogenesis. Interestingly, our adoptive transfer experiments, while confirming the pathogenic function of CD207^+^ DCs, also highlighted the critical importance of this spatial regulation. Following the transfer of in vitro‐generated CD207^+^ BMDCs, we noted a spatial pattern of immune reconstitution that differed from the endogenous condition. The restorative increases in CD8^+^ T‐cell expansion and GZMB expression occurred in the large airways, small airways, and alveoli regions, whereas GZMA expression increased primarily in the alveoli. This spatial mismatch likely arises from the experimental delivery route [55]. Our model utilized an intranasal instillation of in vitro‐differentiated BMDCs, which differs from the continuous, systemic supply of DC precursors under physiological conditions. This delivery method may cause preferential deposition of cells and subsequent T‐cell priming in the larger airways. However, systemic alternatives like intravenous and intraperitoneal injection are limited by poor transfer efficiency to the lung parenchyma, as significant cell trapping in organs such as the liver and spleen prevents sufficient CD207^+^ DC engraftment [56]. Furthermore, the distinct localization of GZMA and GZMB expression suggests that CD8^+^ T‐cell effector functions could be compartmentalized within the lung, as the maturation and homing properties of cultured BMDCs may differ from their endogenous counterparts. Future work should explore how DC origin, differentiation state, and delivery route collectively determine the anatomical landscape of the resulting immune response. Due to the limited availability of large airway samples, we did not include them in direct comparisons in the human study. Although murine models are valuable for investigating immune mechanisms, structural and anatomical differences between mice and humans may limit the direct translation of our findings. Thus, future studies using human lung organoids and primary human cells are necessary to validate and expand upon these results in human‐specific systems. We acknowledge that, although immunohistochemistry is useful for assessing cell density and spatial distribution in lung tissues, the single‐marker identification limits cell specificity, even though we have incorporated multi‐parameter flow cytometry in our in vitro experiments for more accurate cell population identification. In future studies, we aim to collect more fresh lung tissue samples to enable higher‐resolution analyses, and advanced methods such as spatial transcriptomics and mass cytometry are needed to further refine cell characterization and investigate gene and protein expression across distinct anatomical structures within the lung.

Adoptive transfer of CD207^+^ DCs into *CD207^−/−^* mice reversed the attenuation of emphysema, inflammation and CD8^+^ T‐cell expansion induced by O_3_ exposure in CD207‐knockout mice confirming the pathogenic role of CD207^+^ DCs. However, these experiments also revealed a discrepancy between immune activation and tissue pathology. While our adoptive transfer experiments confirmed the pathogenic role of CD207^+^ DCs, they also revealed a discrepancy between immune activation and tissue pathology. While the transfer of CD207^+^ DCs effectively restored CD8^+^ T‐cell expansion and granzyme production in *CD207 ^−/−^* mice, the severity of emphysema showed only a limited correlation with the proportion of engrafted pulmonary CD207^+^ DCs. This suggests that while CD207^+^ DCs are critical for initiating CD8^+^ T‐cell activation, the progression to emphysematous destruction is not solely dependent on the magnitude of this immune response. It is likely governed by more complex regulatory networks that protect against excessive tissue damage [57, 58, 59], which warrants further research to investigate the precise balancing point and regulatory mechanisms.

Recent studies have proposed various innovative treatments for COPD. For instance, Guo et al. prevented emphysema progression by inhibiting the immunoproteasome in polarized macrophages using PLGA nanoparticles [60]. Moreover, Zhu [61], Craparo [62], and their teams have developed targeted pulmonary drug delivery systems based on nanomedicine. Our research highlights targeting CD207^+^ DCs as a potential immunotherapy for emphysema, although its safety and systemic effects remain uncertain. Despite these concerns, *CD207^−/−^* mice did not exhibit compromised airway epithelial and mucus barrier integrity in our animal model. However, in the work by Xu and colleagues, the knockout of CD207 enhanced epicutaneous sensitization in a model of atopic dermatitis [63]. In the mucosal epithelia of the vagina and foreskin, HIV‐1 pathogens are captured and internalized via a CD207‐dependent restricted pathway [64]. Thus, it remains to be determined whether targeting CD207 impacts the mucosal immune response against allergen challenges or pathogens in the airways, potentially increasing susceptibility to infections. Further investigation is required to elucidate the risk‐benefit profile of inhibiting CD207^+^ DC function in COPD.

Several limitations should be noted. First, the observed sex distribution imbalance in both our human participants and mouse model may potentially introduce confounding bias. Our clinical cohort showed a significant underrepresentation of females in both the COPD group and smoker controls, and future studies should aim for sex‐balanced recruitment to address this issue. For our animal experiments, only male mice were used. This decision was based on previous evidence suggesting that estrogen can promote GM‐CSF‐driven differentiation of CD207^+^ DCs from bone marrow progenitors in vitro [65], and that fluctuating estrogen levels may confound anti‐inflammatory effects and introduce variability in immune responses [66]. However, this sex‐specific design limits the applicability of our findings. In future research, we plan to extend our investigations to include female mice and explore potential sex‐specific differences in CD207^+^ DC‐driven immune responses in emphysema. Second, the restricted sample size of large airway specimens limited robust statistical comparisons, as most lung tissues were collected from patients undergoing video‐assisted thoracoscopic surgery (VATS). This surgical approach typically yields peripheral rather than large airway tissue. Although we partially compensated with additional large airway data from animal models, future studies with larger cohorts and balanced airway sampling are needed to build upon our findings. Moreover, culturing primary human cells at an air‐liquid interface with O_3_ exposure, may better mimic the in vivo effects on CD207^+^ DC differentiation.

## 4 Conclusion

In summary, this study establishes CD207^+^ DCs as critical mediators of emphysema pathogenesis. We demonstrate that these cells drive disease progression by promoting CD8^+^ T‐cell cytotoxicity through a novel mechanism involving Birbeck granule‐dependent MHC‐I trafficking for antigen cross‐presentation. Furthermore, we uncover an upstream pathway where cigarette smoke and oxidative stress induce epithelial GM‐CSF production, which in turn drives the expansion of this pathogenic CD207^+^ DC population. These findings fundamentally advance our understanding of emphysema immunopathogenesis and highlight the CD207^+^ DC axis as a promising therapeutic target for COPD management.

## 5 Experimental Methods

### 5.1 Participant Characteristics

The study was approved by the Ethics Committee of the First Affiliated Hospital of Nanjing Medical University (2019‐SR‐371). Participants were categorized as non‐smoker controls (*n* = 35), smoker controls (*n* = 20) and patients with COPD (*n* = 23). Non‐smoker controls were defined as individuals without smoking history. Smoker controls were current smokers without any evidence of airflow limitation. COPD was diagnosed based on the Global Initiative for Chronic Obstructive Lung Disease (GOLD) guidelines (FEV1/FVC < 70%). No participants had a history of asthma. Lung tissue samples were collected from non‐tumorous regions in patients undergoing lung cancer resection. Clinical characteristics of non‐smoker controls, smoker controls, and all COPD participants were summarized in Table 1, where comparisons were performed among the three groups. Detailed subgroup characteristics of COPD participants according to GOLD stages (GOLD 1, 2 and 3 defined by the 2025 GOLD report) [67] were presented in Table 2, with subgroup comparisons performed within the COPD group only.

Definition of abbreviations: M/F, male/female; BMI, body mass index; FEV1%pred, percentage of predicted forced expiratory volume in 1 s; FVC%pred, percentage of predicted forced vital capacity; FEV1/FVC, the ratio of forced expiratory volume in 1 s to forced vital capacity.

Data were shown with mean ± standard deviation (SD). Categorical variables (sex) were analyzed using Fisher's exact test; continuous variables were analyzed using one‐way ANOVA.

Data were shown with mean ± SD. The severity of disease was graded based on post‐bronchodilator FEV1. GOLD stage 1 indicates FEV1 ≥80% predicted; GOLD stage 2 indicates 50% ≤ FEV1 < 80% predicted; GOLD stage 3 indicates 30% ≤ FEV1< 50% predicted. Categorical variables (sex) were analyzed using Fisher's exact test; continuous variables were analyzed using one‐way ANOVA.

### 5.2 Dataset Collection

Four publicly available gene expression datasets were used in this study: GSE47460, GSE5058, GSE8545, and GSE18385. Details for each dataset, including sample grouping, age, gender distribution, and tissue source, were summarized in Table S1. All datasets were based on microarray experiments, where gene expression was measured as fluorescence intensity and reported in arbitrary units (AU). These values were typically log‐transformed for analysis, producing unitless data that represent relative expression levels. Because the GSE5058 and GSE8545 were both derived from small airways and utilized the same microarray platform, we therefore combined them in to a merged dataset. Potential batch effects were removed using the “combat” R package. All data were downloaded from the Gene Expression Omnibus (GEO) database (www.ncbi.nlm.nih.gov/geo/), and were pre‐processed and analyzed according to standard protocols. The GSE47460 dataset was used for functional enrichment analyses. Genes showing significant positive correlation with CD207 expression (Pearson r > 0.4, p < 0.05) were selected for further analysis. Functional annotation, including Gene Ontology (GO) and Kyoto Encyclopedia of Genes and Genomes (KEGG) pathway enrichment, was performed using Metascape. Pathways with a false discovery rate (FDR)‐adjusted p‐values < 0.05 were considered statistically significant.

### 5.3 Ozone (O_3_)‐Induced Emphysema Model

All animal experiments were conducted in accordance with protocols approved by the Institutional Animal Care and Use Committee of Nanjing Medical University (IACUC‐1810021). Male C57BL/6 mice were exposed to O_3_ (3 ppm) for 3 h a day, twice a week for a period of 1, 3 or 6 weeks. Control mice were exposed to normal air. After the last exposure, mice were euthanized for the collection of peripheral blood, bronchoalveolar lavage fluid (BALF), and lung tissue.

### 5.4 *CD207*‐Deficient (*CD207* ^−/−^) Mice

Heterozygous CD207 knockout (*CD207^+/−^*) mice on a C57BL/6 genetic background were kindly provided by Prof. Xu Yao at the Institute of Dermatology, Chinese Academy of Medical Sciences and Peking Union Medical College (originally obtained from the Spanish National Biotechnology Centre), relevant details were available in Prof. Yao's previous work [63]. Mice were housed at the Animal Experimental Center of Nanjing Medical University and maintained under standard conditions (22‐24 °C, 12‐h light/dark cycle). *CD207^−/−^* and wild‐type (WT) littermates were generated by intercrossing heterozygous mice. Genotyping of *CD207^−/−^* and WT mice was performed using DNA extracted from tail biopsies. Allele‐specific PCR primers were used to distinguish between wild‐type and knockout alleles, and the products were analyzed by agarose gel electrophoresis to confirm the genotypes. Primer sequences were provided in Table S2.

### 5.5 Generation of Bone Marrow‐Derived Dendritic Cells (BMDCs)

Bone marrow was harvested from the femurs and tibias of 6‐ to 8‐week‐old male C57BL/6 mice. Briefly, after euthanasia, bones were sterilized in 75% ethanol and washed with sterile PBS. Bone marrow was flushed out with PBS using a syringe, and the resulting cell suspension was filtered through a 200‐mesh nylon screen to remove bone fragments. Following red blood cell lysis, the remaining cells were cultured in RPMI‐1640 medium supplemented with 10% fetal bovine serum, 1% penicillin‐streptomycin, recombinant mouse GM‐CSF (20 ng/mL; R&D Systems, #415‐ML‐005), and IL‐4 (20 ng/mL; R&D Systems, #404‐ML‐010/CF). Half of the culture medium was replaced with fresh, cytokine‐containing medium every 2 days. After 7 days of culture, non‐adherent and loosely adherent cells were collected as immature BMDCs.

### 5.6 Adoptive Transfer

For adoptive transfer experiments, CD207^+^ DCs were isolated from BMDC cultures derived from donor male C57BL/6 mice. Recipient male *CD207^−/−^* mice were lightly anesthetized and intranasally administered with either a low dose (5 × 10^4^ cells) or a high dose (1 × 10^5^ cells) of sorted CD207^+^ DCs suspended in 50 µL of sterile PBS. Cell transfer was performed 24 h prior to the initiation of O_3_ exposure. Control *CD207^−/−^* mice received an equal volume of sterile PBS. Successful engraftment was confirmed by flow cytometric analysis of lung tissue 24 h post‐transfer.

### 5.7 Alveolar Mean Linear Intercept (MLI)

The severity of emphysema was quantified by measuring the alveolar MLI in H&E‐stained lung tissue sections. MLI was calculated by dividing the total length of a grid of lines drawn over a lung parenchyma image by the total number of alveolar walls that intercepted those lines.

### 5.8 Inflammation Score

The severity of inflammatory cell infiltration in H&E‐stained lung tissue was assessed using a semi‐quantitative scoring system. Scores were assigned as follows: 0, absence of inflammatory cells; 1, presence of scattered, sparse inflammatory cells; 2, a single layer of inflammatory cells forming a ring around airways or vessels; 3, a ring of inflammatory cells two to four layers deep; and 4, a ring of inflammatory cells more than four layers deep.

### 5.9 Immunohistochemistry Staining

Formalin‐fixed, paraffin‐embedded lung sections were baked for 2 h at 65°C, deparaffinized, and rehydrated. Antigen retrieval was performed by heating slides in a pressure cooker for 10 min in citrate buffer (pH 6.0; MXB Biotechnologies, MVS‐0066). Endogenous peroxidase activity was blocked by incubating sections in 3% H_2_O_2_ for 15 min. Non‐specific binding was blocked with 10% goat serum for 1 h at room temperature. Sections were then incubated with primary antibodies (diluted in blocking solution) overnight at 4°C. The following primary antibodies were used: CD207 (Novus, #DDX0362P‐100), CD8α (Invitrogen, #14‐0081‐82), CD4 (Invitrogen, #11‐0041‐82), CD19 (Invitrogen, #14‐0194‐82), GZMA (Sigma‐Aldrich, #HPA054134), GZMB (Sigma‐Aldrich, #HPA003418), and GM‐CSF (Abcam, #ab316862). The following day, sections were incubated with the appropriate horseradish peroxidase (HRP)‐conjugated secondary antibodies, and the signal was developed using a 3,3'‐diaminobenzidine (DAB) substrate kit. Sections were washed three times with PBS between each step. Isotype‐matched negative controls were included for all experiments. For humans, small airways were defined as structures with a basement membrane (BM) perimeter ≤ 6 mm [68], while in mice, small airways were identified as those with a short diameter ≤ 90 µm [69]. 3–6 small airways were analysed per sample, and the density of the inflammatory cells was quantified in each airway. Cells were classified in airway if immunostaining localized to the bronchiolar epithelium or submucosa. For the alveolar parenchyma analyses, 8–10 fields per sample were selected. Cells were classified alveolar if they were located within the alveolar septa or distal alveolar sacs. To prevent overlap between compartments, cells within 200 µm of the airway wall were excluded from the alveolar assessment. Regions with particular lymphoid aggregates or inflamed foci should be carefully avoided. All statistical analyses were performed at 400× magnification. Image ProPlus 6.0 software was utilized for image analysis. The immunostaining quantification was standardized as follows: surface markers (CD8, CD207, CD4 and CD19) were quantified by positive cells counted and normalized to the length of small airway basement membranes or alveolar septa (cells/mm), respectively [70]. Secreted effector proteins (GZMA, GZMB and GM‐CSF) were quantified by immunodensity calculated as average optical density (AOD).

### 5.10 Preparation of Cigarette Smoke Extract (CSE)

Research cigarettes (11 mg tar, 1.2 mg nicotine) were used to generate CSE. The filter of one cigarette was connected via tubing to a flask containing 10 mL of serum‐free cell culture medium. Smoke was bubbled through the medium by drawing it with a 50 mL syringe at a constant rate until the cigarette was fully consumed. This solution, defined as 100% CSE, was then sterile‐filtered through a 0.22 µm membrane.

### 5.11 Hydrogen Peroxide (H_2_O_2_) Treatment

A stock solution of 8.8 M H_2_O_2_ was prepared by diluting 30% (w/w) H_2_O_2_ in sterile PBS. This stock was then sterile‐filtered (0.22 µm) and further diluted in cell culture medium to a final working concentration of 100 µM for cell treatments.

### 5.12 Isolation of Naïve CD8^+^ T Cells and Mixed Lymphocyte Reaction (MLR)

Spleens were harvested from C57BL/6 mice and processed into a single‐cell suspension in PBE buffer (PBS supplemented with 0.5% BSA and 2 mM EDTA, pH 7.2). Naïve CD8^+^ T cells were isolated from this suspension via positive selection using a magnetic‐activated cell sorting (MACS) system (Miltenyi Biotec). Briefly, cells were incubated with CD8α MicroBeads (Miltenyi Biotec, #130‐117‐044) and passed through an LS Column in a magnetic field. The magnetically retained CD8^+^ T cells were eluted, washed, and resuspended in complete RPMI‐1640 medium (supplemented with 10% FBS and 1% penicillin‐streptomycin). For the MLR, these purified naïve CD8^+^ T cells were co‐cultured with the previously generated BMDCs at a 4:1 T cell‐to‐BMDC ratio in a humidified incubator at 37°C and 5% CO_2_.

### 5.13 Quantitative Real‐time PCR

Total RNAs of lung tissue, BMDC and 16HBE were extracted using TRIzol reagent (Invitrogen, #15596018). Reverse transcription and cDNA synthesis were carried out using a reverse transcription kit (Takara, #RR047A). The primer sequences of the genes of interest were designed using primer design software, and primer specificity was determined based on the BLAST alignment in NCBI. The primers were synthesized by Shanghai Shenggong Bioengineering Co., Ltd., and the sequences were listed in Table S2. qPCR was performed using ChamQ Universal SYBR qPCR Master Mix (Vazyme). The results were calculated and quantified based on the 2 ^−ΔΔCt^ method, and GAPDH was used as an internal reference.

### 5.14 Transmission Electron Microscopy (TEM)

Induced BMDCs were centrifuged and fixed in 2.5% glutaraldehyde. Subsequently, the fixed cells were post‐fixed with 1% osmium tetroxide (Electron Microscopy Sciences, #19100) in deionized water for 1 h at room temperature. Following this, the cell pellets were treated with 1% uranyl acetate for 1 h and then dehydrated through graded ethanol additions, each for 5 min. Finally, the samples were embedded in Eponate resin containing NMA, DDSA, and DMP‐30. The embedded pellets were sectioned into 70 nm ultrathin sections and imaged using a JEOL‐1010 transmission electron microscope.

### 5.15 Statistical Analysis

Data were shown as the mean ± SD or SEM. All statistical analyses were performed with R Statistical Software (v4.4.2; R Core Team). Statistical analyses used the Shapiro‐Wilk test to test for normality. Analysis between two groups used unpaired Student's t test for normally distributed parameters and the Wilcoxon rank sum test for non‐normally distributed parameters. One‐way or two‐way ANOVA was used to assess the statistical significance. One‐way ANOVA with Tukey's post hoc test and Dunnett's post hoc test were conducted for multiple comparisons when applicable. Spearman's correlation analysis was used to determine the correlation coefficient (r) between two groups and corresponding p values. Proportions of variable among groups were compared using the Chi‐square test or Fisher's exact test, as appropriate. A two‐tailed p‐value <0.05 was considered statistically significant for all hypothetical tests.

## Author Contributions

X.Y., X.Z., and M.J. contributed to conceptualization, methodology, and funding acquisition. S.X., Y.W., and F.L. were responsible for data acquisition and manuscript drafting. S.X., Y.W., and H.F. conducted animal breeding, experimental design, and data analysis. Y.M., Y.O., and X.Y. provided technical support, reagents, and experimental assistance. I.M.A. contributed to experimental design, and manuscript review, and revision. All authors have read and approved the final version of the manuscript. The authorship order, including the designation of two co–first authors, was determined through mutual discussion based on their respective contributions.

## Funding

This work was supported by theNational Natural Science Foundation of China [grant numbers 82170040, 81870039, 82200035, and 82370035]; the Natural Science Foundation of Jiangsu Province [grant number BK20210957].

## Conflicts of Interest

All authors declare no conflict of interest.

## Supporting information




**Supporting File**: advs73702‐sup‐0001‐SuppMat.docx.

## Data Availability

The transcriptome data of human lung tissues analyzed in this study are available in the GEO repository (https://www.ncbi.nlm.nih.gov/geo/) under accession code GSE47460, GSE5058, GSE8545 and GSE18385. All other data generated or analyzed during this study are included in this published article and its supporting information files. Further inquiries can be directed to the corresponding author upon reasonable request.
