# Supplementary material for: CD207‐Positive Dendritic Cells Promote Emphysema Through CD8+ T Cell Pathway in Chronic Obstructive Pulmonary Disease
Source: Adv Sci (Weinh). 2026 Jan 7;13(16):e12993. doi: 10.1002/advs.202412993 (PMC13042610; doi:10.1002/advs.202412993)
Supplement: Supplementary file 1 — Supporting File: advs73702‐sup‐0001‐SuppMat.docx. [file ADVS-13-e12993-s001.docx]

**Supporting Information**

**Table S1.** **Characteristics of the Included GEO Datasets**

**Table S1-1.** Cohort Characteristics of GSE47460 Dataset

|  | COPD |
| --- | --- |
| Number (n) | 145 |
| Age (years) | 65.90 ± 9.11 |
| Sex (M/F) | 79/66 |
| Smoking status (%) |  |
| Current | 11 (7.6) |
| Ever | 125 (86.2) |
| Never | 9 (6.2) |
| %emphysema | 13.79 ± 15.60 |
| pre-BD FEV1%pred | 52.53 ± 23.01 |
| post-BD FEV1%pred | 55.57 ± 22.23 |
| pre-BD FVC%pred | 75.42 ± 17.75 |
| post-BD FVC%pred | 80.89 ± 17.40 |
| DLCO%pred | 56.56 ± 22.27 |

Definition of abbreviations: M/F, male/female; %emphysema, percentage of lung pixels less than −950 Hounsfield units; BD, bronchodilator; FEV1%pred, forced expiratory volume in 1s percentage predicted; FVC%pred, forced vital capacities percentage predicted; DLCO% pred, diffusing capacity of the lungs for carbon monoxide percentage predicted. Data are shown with mean ± standard deviation (SD).

**Table S1-2.** Cohort Characteristics of GSE5058 & GSE8545 Datasets

|  | Non-smoker | Smoker | COPD | p |
| --- | --- | --- | --- | --- |
| Number (n) | 19 | 21 | 21 |  |
| Age (year) | 41.32 ± 6.89 | 45.62 ± 5.32 | 49.33 ± 6.49 | 0.001 |
| Sex (M/F) | 15/4 | 14/7 | 17/4 | 0.511 |
| Ethnic (%) |  |  |  | 0.284 |
| black | 10 (52.6) | 12 (57.1) | 8 (38.1) |  |
| hispanic | 3 (15.8) | 0 (0.0) | 4 (19.0) |  |
| white | 6 (31.6) | 9 (42.9) | 9 (42.9) |  |

Data are shown with mean ± SD. Categorical variables were analyzed using Fisher’s exact test; continuous variables were analyzed using one-way ANOVA.

**Table S1-3.** Characteristics of Large Airway Samples in the GSE18385 Dataset

|  | Non-smoker | Smoker | p |
| --- | --- | --- | --- |
| Number (n) | 20 | 32 |  |
| Age (year) | 40.25 ± 7.80 | 43.75 ± 6.46 | 0.086 |
| Sex (M/F) | 14/6 | 22/10 | 1 |
| Ethnic (%) |  |  | 0.4 |
| asian | 1 (5.0) | 0 (0.0) |  |
| black | 9 (45.0) | 20 (62.5) |  |
| black/hispanic | 1 (5.0) | 0 (0.0) |  |
| hispanic | 3 (15.0) | 4 (12.5) |  |
| white | 6 (30.0) | 8 (25.0) |  |

Data are shown with mean ± SD. Categorical variables were analyzed using Fisher’s exact test or Chi-square test; continuous variables were analyzed using student’s two-tailed t-test.

**Table S1-4.** Characteristics of Small Airway Samples in the GSE18385 Dataset

|  | Non-smoker | Smoker | p |
| --- | --- | --- | --- |
| Number (n) | 51 | 58 |  |
| Age (year) | 41.24 ± 11.45 | 42.90 ± 7.25 | 0.362 |
| Sex (M/F) | 36/15 | 38/20 | 0.719 |
| Ethnic (%) |  |  | 0.221 |
| asian | 1 (2.0) | 0 (0.0) |  |
| black | 23 (45.1) | 35 (60.3) |  |
| black/hispanic | 1 (2.0) | 0 (0.0) |  |
| hispanic | 6 (11.8) | 9 (15.5) |  |
| white | 20 (39.2) | 14 (24.1) |  |

Data are shown with mean ± SD. Categorical variables were analyzed using Fisher’s exact test or Chi-square test; continuous variables were analyzed using student’s two-tailed t-test.

**Table S2.** List of Primer Sequences Utilized in This Study

| Mouse CD86 | Forward | 5’-GTCACAAGAAGCCGAATCA-3’ |
| --- | --- | --- |
|  | Reverse | 5’-GGGGTTCAAGTTCCTTCAG-3’ |
| Mouse CD80 | Forward | 5’-GCCTTGCCGTTACAACTC-3’ |
|  | Reverse | 5’-TACTCGGGCCACACTTTT-3’ |
| Mouse CD40 | Forward | 5’-GACTGCTTGCTGACCTTTG-3’ |
|  | Reverse | 5’-AGCTCTCCCTCCATCCTT-3’ |
| Mouse MHC-I (*H2-K1*) | Forward | 5’-GACCCATCACAGCAGACC-3’ |
|  | Reverse | 5’-GCTCCTCCCCATTCAACT-3’ |
| Mouse PU.1 (*Spi1*) | Forward | 5’-ACAGCATCTGGTGGGTGGAC-3’ |
|  | Reverse | 5’-GCCTGTCTTGCCGTAGTTGC-3’ |
| Mouse Runx3 | Forward | 5’-TATCCCTCTCTGGGCCTTCT-3’ |
|  | Reverse | 5’-GGAAACTGAGTCCAGCCAAG-3’ |
| Mouse GM-CSF (*CSF2*) | Forward | 5’-TGCGGATTTCATAGACAGC-3’ |
|  | Reverse | 5’-TCATTACGCAGGCACAAA-3’ |
| Human GM-CSF (*CSF2*) | Forward | 5’-GCATGTAGAGGCCATCAAAGA-3’ |
|  | Reverse | 5’-CGGGTCTGCACACATGTTA-3’ |
| Mouse GAPDH | Forward | 5’-CCTCCTCCAATTCAACCCT-3’ |
|  | Reverse | 5’-CACCGACCTTCACCATTTT-3’ |
| Mouse CD207 | Forward WT | 5’-CATTTGTAGGGTGGGTGGATAGAT-3’ |
|  | Reverse WT | 5’-GTTTGTCCACTGTGAAGTGCGC-3’ |
|  | Reverse CRE | 5’-ACATTGGATATCCTCCTGTGACCT-3’ |

**Figure S1**


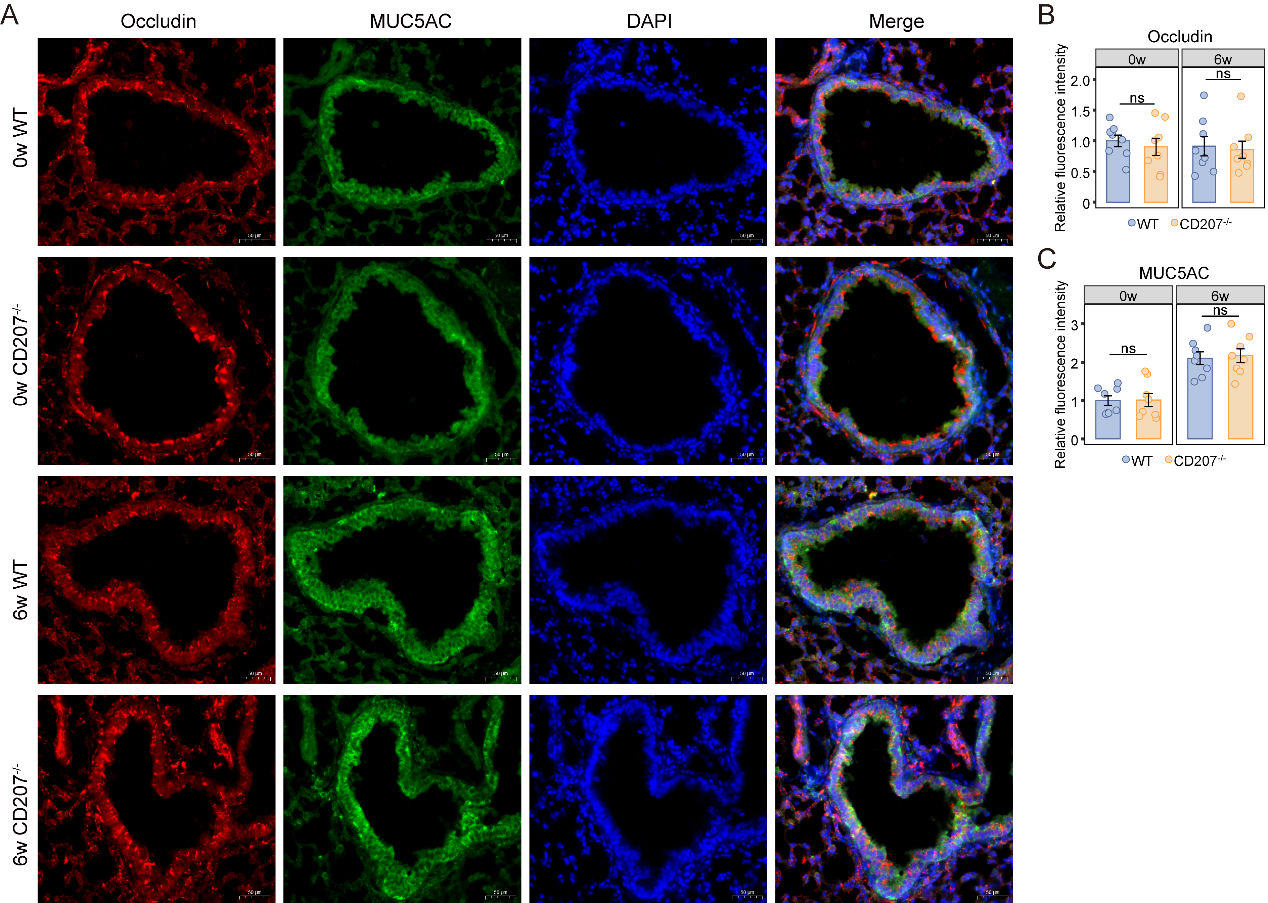


**Figure S1.** A) Representative immunofluorescence staining of Occludin (red) and MUC5AC (green) in airways from WT and *CD207^-/-^* mice after 0 and 6 weeks of ozone exposure. Nuclei are counterstained with DAPI (blue). Scale bar: 50 μm. B) Quantification of Occludin fluorescence intensity normalized to the 0-week WT group. C) Quantification of MUC5AC fluorescence intensity normalized to the 0-week WT group. Data are presented as mean ± SEM (n = 8 per group). Statistical significance was determined by two-tailed Student’s t-test.

**Figure S2**

**
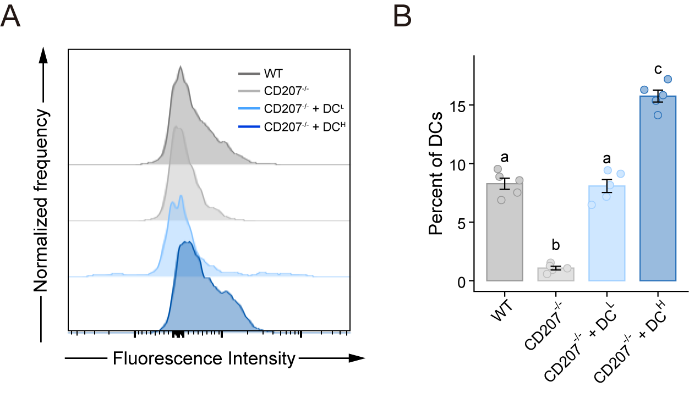
**

**Figure S2.** Verification of CD207⁺ DC engraftment following adoptive transfer. A) The four experimental groups: WT, *CD207^-/-^*, and *CD207^-/-^* mice that received an adoptive transfer of low-dose (*CD207^-/-^* + DC^L^) or high-dose (*CD207^-/-^* + DCᴴ) CD207⁺ DCs. Representative flow cytometry histograms showing the abundance of CD207⁺ DCs within the total lung DC population 24 hours after intranasal administration. Quantification of the frequency of CD207⁺ DCs as a percentage of the total lung DC population. Data are presented as mean ± SEM (n = 5 per group). Statistical significance was determined by one-way ANOVA with Tukey's post hoc test. Different letters (a, b, c) indicate statistically significant differences between groups (p < 0.05).

**Figure S3**


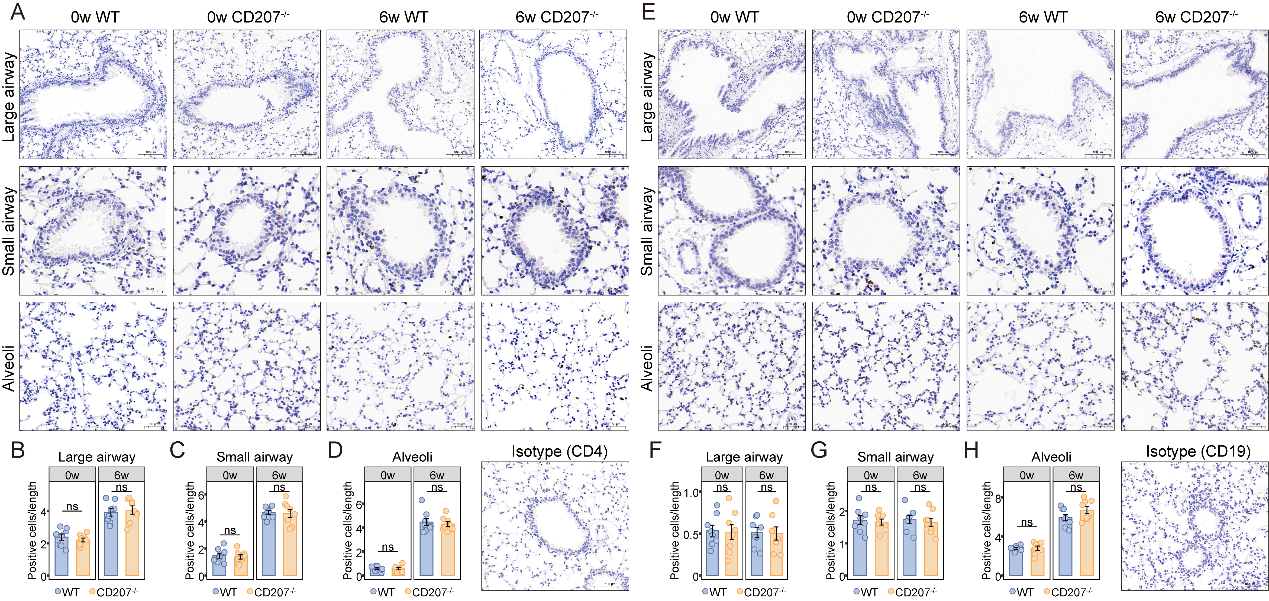


**Figure S3.** A) Representative CD4 immunohistochemical staining in large airway, small airway and alveolar regions of WT and *CD207^-/-^* emphysema model. Scale bars are shown in each panel. B-D) Quantification of CD4-positive cells normalized to basement membrane or alveolar septa length (cells/mm) in each region. E) Representative CD19 staining in large airway, small airway and alveolar regions of WT and *CD207^-/-^* emphysema model. Scale bars are shown in each panel. F-H) Quantification of CD19-positive cells normalized to basement membrane or alveolar septa length (cells/mm) in each region. Data are presented as mean ± SEM. Statistical significance was determined by two-tailed Student’s t-test.

**Figure S4**


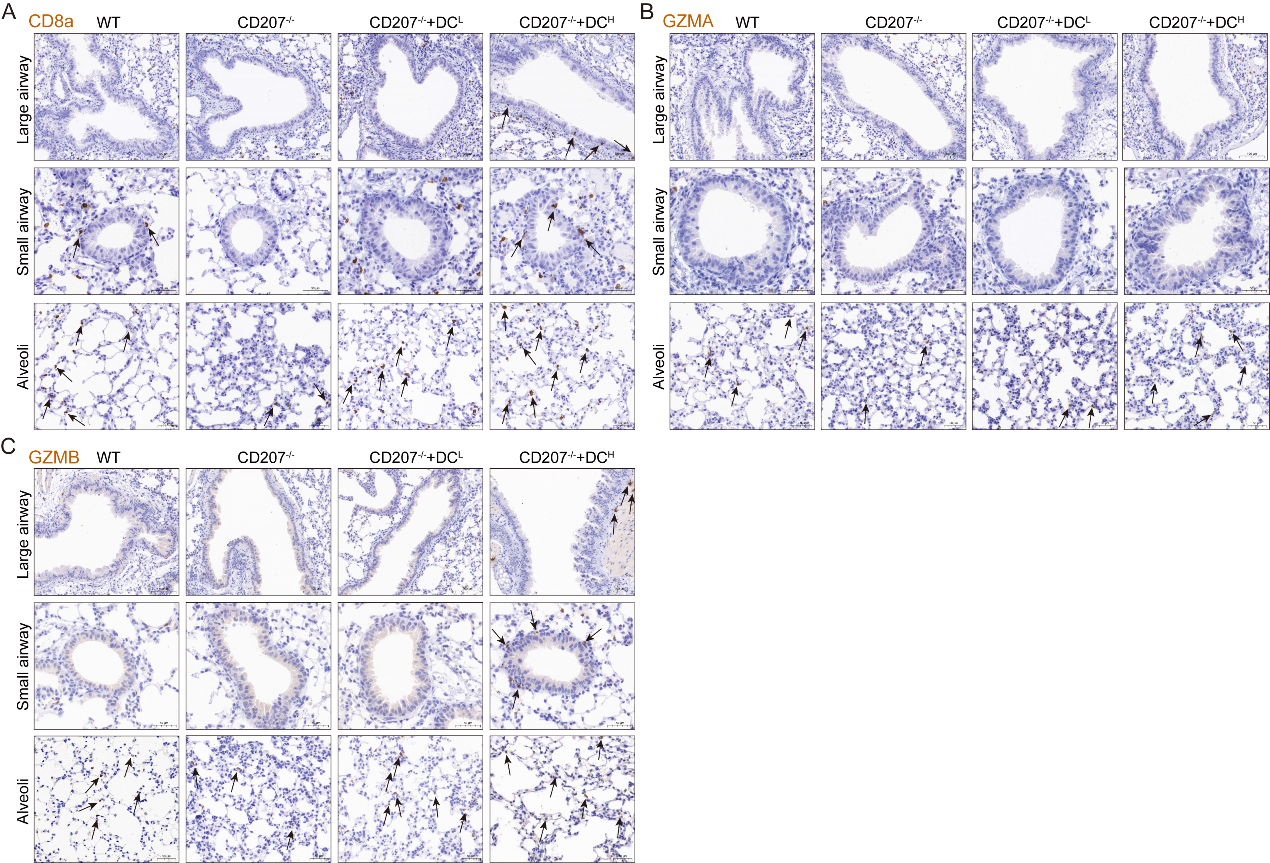


**Figure S4.** A) Representative immunohistochemical staining for CD8α in large airways, small airways and alveolar regions from WT, *CD207^-/-^*, and *CD207^-/-^* mice that received an adoptive transfer of low-dose (*CD207^-/-^* + DC^L^) or high-dose (*CD207^-/-^* + DCᴴ) CD207⁺ DCs after 6 weeks O_3_ exposure. Scale bars are shown in each panel. Original magnification: 200× (large airways), 400× (small airways), 300× (alveolar regions). B) Representative immunohistochemical staining for GZMA in large airways, small airways and alveolar regions from WT, *CD207^-/-^*, *CD207^-/-^* + DC^L^ and *CD207^-/-^* + DCᴴ groups. Scale bars are shown in each panel. Original magnification: 200× (large airways), 400× (small airways), 300× (alveolar regions). C) Representative immunohistochemical staining for GZMB in large airways, small airways and alveolar regions from WT, *CD207^-/-^*, *CD207^-/-^* + DC^L^ and *CD207^-/-^* + DCᴴ groups. Scale bars are shown in each panel. Original magnification: 200× (large airways), 400× (small airways), 300× (alveolar regions).

**Figure S5**


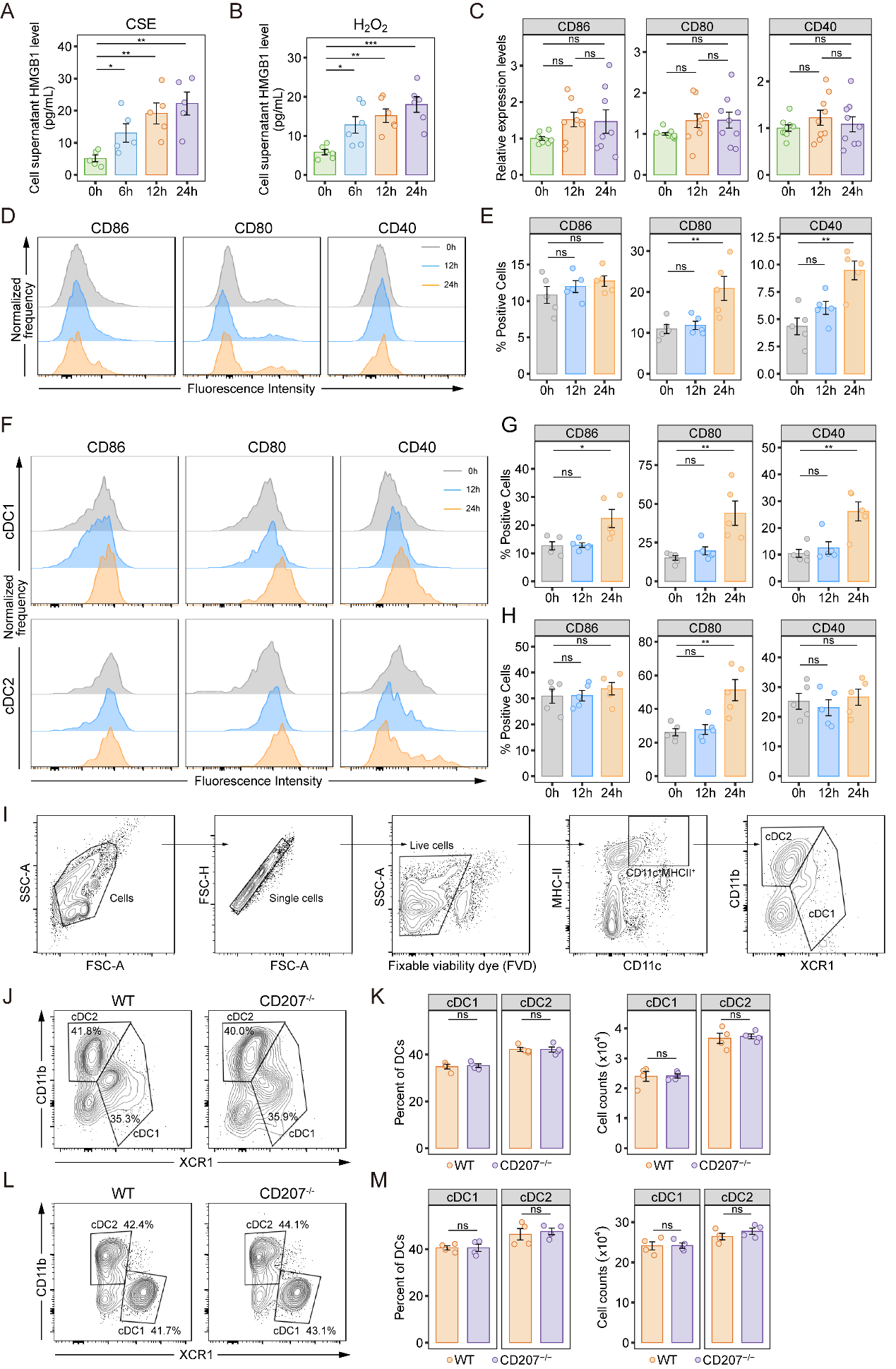


**Figure S5.** A) HMGB1 levels in the supernatant of 16HBE cells treated with 5% CSE for 0, 6, 12, and 24 hours measured by ELISA (n = 5 per group). B) HMGB1 HS level in supernatants from 16HBE cells treated with 100 μM H_2_O_2_ for the same time periods (n = 6 per group). C) Relative mRNA expression of CD86, CD80, and CD40 in CD207^+^ DCs treated with HMGB1 for 0, 12, and 24 hours (n = 8-9 per group). D) Representative flow cytometry histograms showing fluorescence intensity of CD86, CD80, and CD40 in CD207⁺ DCs (n = 5 per group). E) Frequency of CD86^+^, CD80^+^, and CD40^+^ cells among CD207⁺ DCs (thresholds defined by FMO controls). F) Representative flow cytometry histograms showing fluorescence intensity of CD86, CD80, and CD40 in cDC1 and cDC2 subsets stimulated with HS (n = 5 per group). G) Frequency of CD86^+^, CD80^+^, and CD40^+^ cells among cDC1 subset. H) Frequency of CD86^+^, CD80^+^, and CD40^+^ cells among cDC2 subset. I) Representative gating strategy for flow cytometric analysis of cDC1 and cDC2 subsets. J) Representative flow cytometry dot plots showing the expression of cDC1 and cDC2 in lung tissues from WT and *CD207^-/-^* mice(n = 4 per group). Numbers adjacent to outlined gates indicate the percentage of cells within each gate relative to the parent population. K) Frequencies (left panel) and absolute numbers (right panel) of cDC1 and cDC2 populations in WT and *CD207^-/-^* lungs. L) Representative flow cytometry dot plots showing the expression of cDC1 and cDC2 in WT and *CD207^-/-^* BMDCs (n = 4 per group). M) Frequencies (left panel) and absolute numbers (right panel) of cDC1 and cDC2 populations in WT and *CD207^-/-^* BMDCs. Data are presented as mean ± SEM. Statistical analysis was performed using a one-way ANOVA with Dunnett’s post-hoc test (A, B, E), one-way ANOVA with Tukey’s post hoc test (C), or two-tailed Student’s t-test (G, H, K, M). *p < 0.05, **p < 0.01, ***p < 0.001.

**Figure S6**


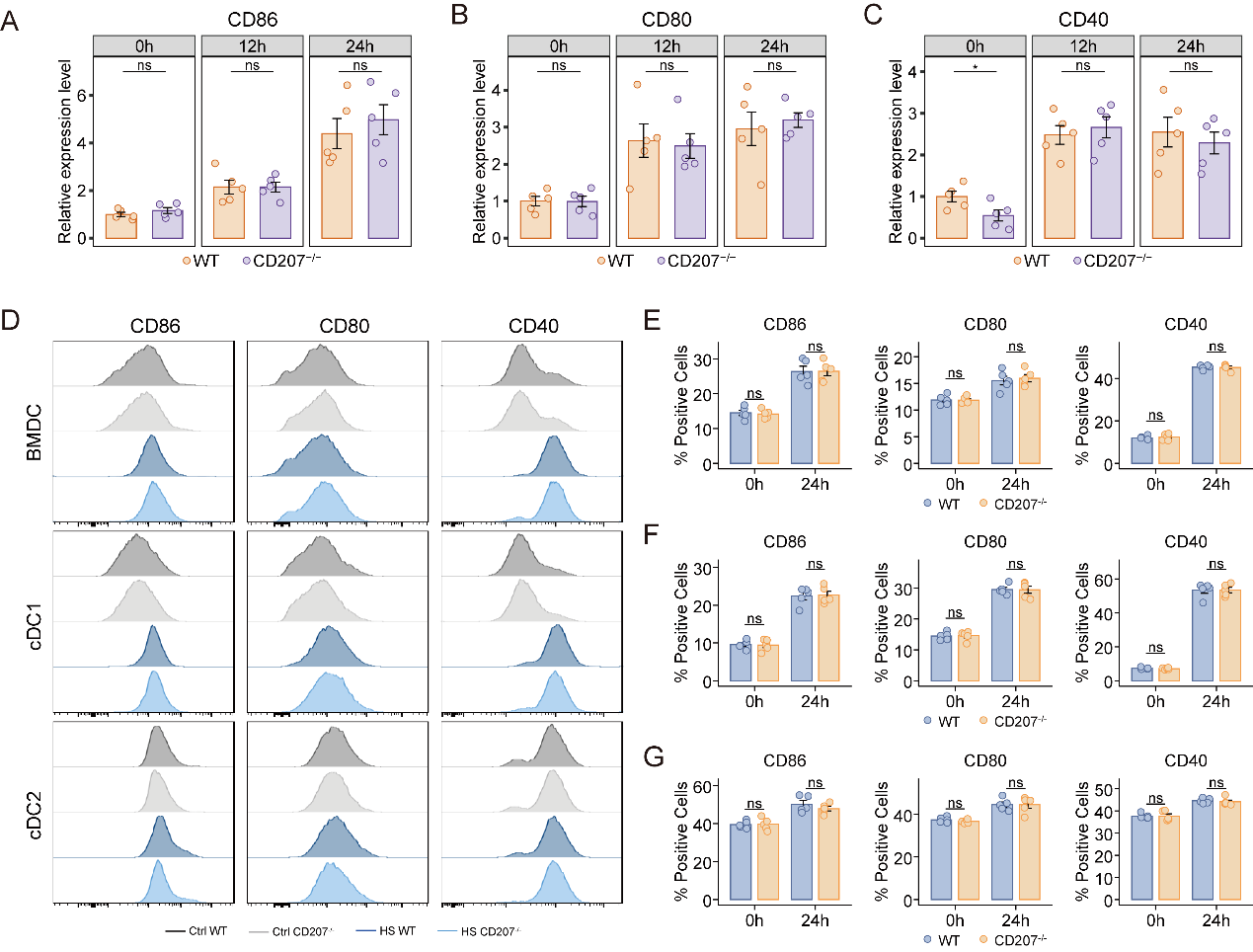


**Figure S6.** A) Relative mRNA expression of CD86 in WT and *CD207^-/-^* BMDCs treated with HS for 0, 12, and 24 hours (n = 5 per group). B) Relative mRNA expression of CD80 in WT and *CD207^-/-^* BMDCs treated with HS for 0, 12, and 24 hours (n = 5 per group). C) Relative mRNA expression of CD40 in WT and *CD207^-/-^* BMDCs treated with HS for 0, 12, and 24 hours (n = 5 per group). D) Representative flow cytometry histograms showing fluorescence intensity of CD86, CD80, and CD40 in BMDCs, cDC1 and cDC2 (n = 5 per group). E-G) Frequency of CD86⁺, CD80⁺, and CD40⁺ cells among total BMDCs (E), the cDC1 subset (F), and the cDC2 subset (G). Data are presented as mean ± SEM. Statistical analysis was performed using two-tailed Student’s t-test. *p < 0.05.

**Figure S7**

**
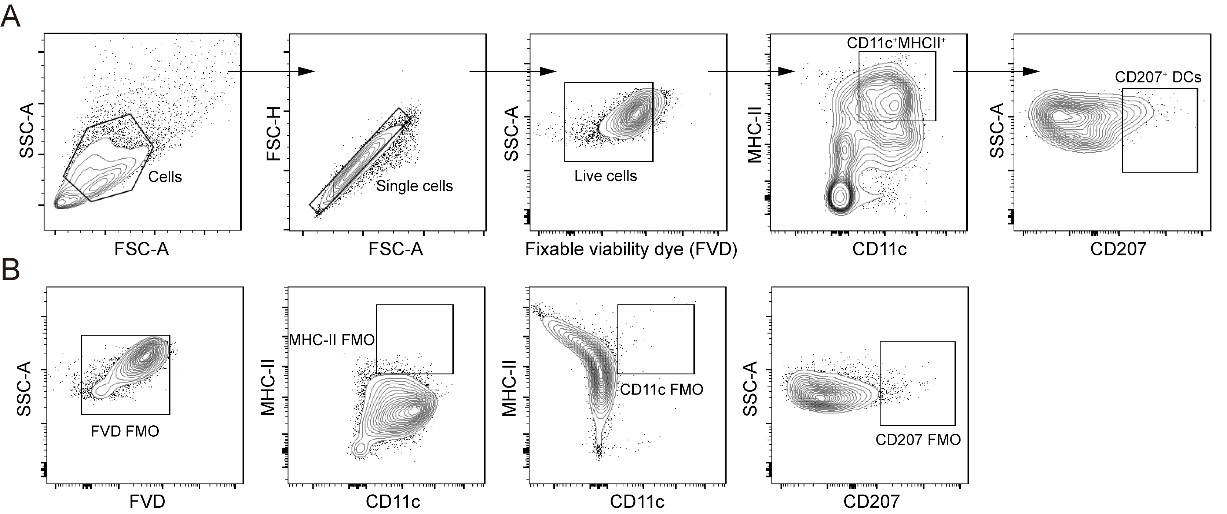
**

**Figure S7.** A) Representative gating strategy for flow cytometric analysis of CD207^+^ DCs. B) Representative dot plots of fluorescence minus one (FMO) controls for gating.
